# Mechanistic Advancements and Translational Progress in Hyaluronic Acid-Based Scaffolds and Conduits for Peripheral Nerve Regeneration

**DOI:** 10.3390/jfb17010014

**Published:** 2025-12-25

**Authors:** Caroline J. Cushman, Naveen A. Sakthiyendran, Maryam Salimi, Evan J. Hernandez, Ruthvik Allala, Tammam Hanna, Anceslo Idicula, Brendan J. MacKay

**Affiliations:** 1 School of Medicine, Texas Tech University Health Sciences Center, Lubbock, TX 79430, USA; 2 Department of Neurological Surgery, Boston University Medical Center, Boston, MA 02115, USA; 3 Department Orthopaedic Surgery, University of Texas Health Science Center, Houton, TX 77030, USA; 4 Department of Health Sciences, College of Health Sciences, Rush University, Chicago, IL 60612, USA; 5 Department of Orthopaedic Surgery, Texas Tech University Health Sciences Center, Lubbock, TX 79430, USAtammam.hanna@ttuhsc.edu (T.H.);

**Keywords:** peripheral nerve repair, hyaluronic acid, nerve biomaterials, nerve regeneration, hyaluronic acid scaffolds

## Abstract

Peripheral nerve injuries often recover poorly. Hyaluronic acid (HA) biomaterials, with regenerative and anti-fibrotic properties, may augment repair. We performed a PRISMA-guided systematic review of PubMed, Scopus, Web of Science, and Embase (January 2000–August 2024), capturing in vitro, in vivo, and clinical investigations of HA in peripheral nerve repair; data on study context, interventions, and outcomes were extracted. Screening and extraction were performed in duplicate. Forty-eight studies met inclusion criteria. Across in vitro and in vivo models, HA-based biomaterials consistently reduced perineural fibrosis, enhanced axonal regeneration, and improved SFI, CMAP, and NCV compared with conventional repair. Several HA hydrogels and composite conduits achieved functional outcomes approaching autografts, particularly when combined with exosomes, neurotrophic factors, or mechanobiologically tuned scaffolds. Early clinical studies demonstrated safety but remain limited by size and short follow-up. Overall, HA-containing biomaterials appear anti-fibrotic, neuroprotective, and pro-regenerative, supporting their promise as adjuncts for peripheral nerve reconstruction. For this to translate into clinical practice, future work should standardize formulations and dosing, employ rigorous, clinically relevant animal models with long-term endpoints, and advance well-powered, controlled trials to test effectiveness and durability in patients. Clinically, HA platforms show promise as anti-adhesion barriers after neurolysis and as biofunctional fillers/coatings for nerve conduits, but standardized formulations and adequately powered trials are needed to define indications and dosing.

## 1. Introduction

Peripheral nerve injuries (PNIs) are common after blunt and penetrating trauma, iatrogenic injury, and tumor resection, leading to chronic pain, sensory loss, weakness, and disability that impair return to work and quality of life [1]. Despite advances in microsurgical technique, tension-free neurorrhaphy, autografting, processed allografts, and conduit-assisted repair, functional outcomes remain heterogeneous, particularly for long gaps, delayed presentation, or scarring/revision contexts [1]. Limitations of current strategies include donor-site morbidity and limited graft length (autograft), immunologic and cost considerations (allograft), and suboptimal bioactivity or mechanical mismatch in some off-the-shelf conduits [1,2,3,4,5]. These gaps have catalyzed interest in biomaterial systems that can (i) physically guide regenerating axons, (ii) modulate the perineural immune and fibrotic milieu, and (iii) deliver bioactive cues with spatial and temporal control [2,3,4,6,7,8,9].

Among candidate materials, hyaluronic acid (HA), a linear, non-sulfated glycosaminoglycan composed of repeating D-glucuronic acid and *N*-acetyl-D-glucosamine, stands out for its biocompatibility, biodegradability, hydrophilicity, and viscoelasticity [3,4]. Endogenous HA is a core extracellular matrix component that regulates hydration, lubrication, and cell–matrix signaling through receptors such as CD44 and RHAMM, and via crosstalk with toll-like receptors [2]. In peripheral nerve biology, these interactions influence Schwann cell migration and alignment, macrophage polarization, angiogenesis, and matrix remodeling, all central to Wallerian degeneration and axonal regrowth [4,10,11]. HA can be formulated as films/wraps (anti-adhesion barriers), injectable hydrogels (native or crosslinked, e.g., HAMA), nerve guidance conduit (NGC) coatings/fillers, or composite scaffolds with chitosan, collagen, PLGA/PLA, β-TCP, or conductive/photothermal nanoparticles [2,4,6,8,9,12,13,14,15,16,17]. Chemical modification (e.g., methacrylation, thiolation) tunes crosslink density, degradation kinetics, porosity, and stiffness, parameters that are increasingly recognized as mechanobiologic regulators of Schwann cell behavior and axon extension [2,4,6,8,9,12,13,14,15,16,17].

HA’s anti-fibrotic and anti-adhesion profile is particularly attractive in revision surgery and neurolysis, where perineural scarring restricts nerve gliding and impedes reinnervation [18,19,20,21]. As a highly hydrated, low-friction interface, HA can reduce fibroblast ingress and collagen deposition while preserving epineurial glide [2]. Beyond serving as a passive barrier, HA hydrogels and composites enable drug, growth factor, or extracellular vesicle (exosome) depots, supporting sustained, localized delivery of neurotrophic and immunomodulatory signals [2]. Early preclinical studies show that HA-containing systems can enhance axon density and diameter, myelin thickness (g-ratio), electrophysiology (CMAP/NCV), and gait metrics (SFI), with several platforms approaching autograft-level performance in short- to mid-term follow-up [6,12,14,15,16,22,23,24,25,26]. Emerging strategies such as granular or anisotropic hydrogels for topographic guidance, magnetically templated alignment, or photothermal anti-adhesion illustrate how HA can be engineered to add directional cues and on-demand bioactivity [6,7,8,9,12,13,14,15,16,23,24,25,26].

At the same time, important translational considerations remain. HA molecular weight and concentration influence viscosity, residence time, receptor engagement, and degradability, while endogenous hyaluronidases in injured tissue can alter persistence and rheology [9,11,14,15,27]. Mechanical integrity of purely HA-based scaffolds may be insufficient for critical-size defects, prompting composite designs to balance compliance (for gliding and mechanosensing) with structural stability (for gap bridging) [9,11,14,15,27] Clinically, HA has seen use as an adhesion barrier after neurolysis or revision carpal tunnel procedures and as a lumen filler/coating to biofunctionalize cleared conduits; however, published human data remain limited by small cohorts, short follow-up, and heterogeneous formulations and outcome measures [2]. Standardization of HA chemistry, dosing, and indications, paired with clinically relevant animal models and well-powered, controlled trials, is necessary to define real-world effectiveness and durability.

In this PRISMA-guided systematic review, we (1) catalog HA-based biomaterial formulations (films, hydrogels, NGCs, composites) used for PNI; (2) synthesize functional (SFI, CMAP, NCV) and histologic outcomes versus conventional repair; (3) highlight medical applications (anti-adhesion after neurolysis, gap repair adjuncts, local therapeutic depots); and (4) identify methodologic and translational gaps, including formulation standards, dosing, mechanobiologic parameters, and study design, needed to accelerate clinical adoption.

## 2. Materials and Methods

### 2.1. Protocol and Search Strategy

This systematic review adhered to PRISMA 2020 guidelines, and the PRISMA checklist is provided in Supplementary File S1. A comprehensive literature search of PubMed, Scopus, Web of Science, and Embase was conducted for studies published between 1 January 2000 and 15 August 2024. This systematic review was prospectively registered with the International Prospective Register of Systematic Reviews (PROSPERO). The review is listed under the title “Hyaluronic Acid-Based Biomaterials in Peripheral Nerve Regeneration: A Systematic Review” (PROSPERO Record ID: 1230404). Search terms combined controlled vocabulary and free-text keywords related to hyaluronic acid and peripheral nerve repair, such as “hyaluronic acid,” “hyaluronan,” “HAMA,” “peripheral nerve,” “nerve regeneration,” “conduit,” and “hydrogel”.

### 2.2. Eligibility Criteria

Studies were eligible if they evaluated hyaluronic acid-based biomaterials in peripheral nerve injury models and reported functional, electrophysiologic, histologic, or adhesion-related outcomes. Eligible designs included in vitro studies, in vivo animal models, randomized controlled trials, cohort studies, and case series. Studies were excluded if they involved central nervous system models, were not published in English, were review articles or conference abstracts, or lacked full-text availability. Studies examining biomaterials without hyaluronic acid components were also excluded.

### 2.3. Study Selection

**Figure 1:**
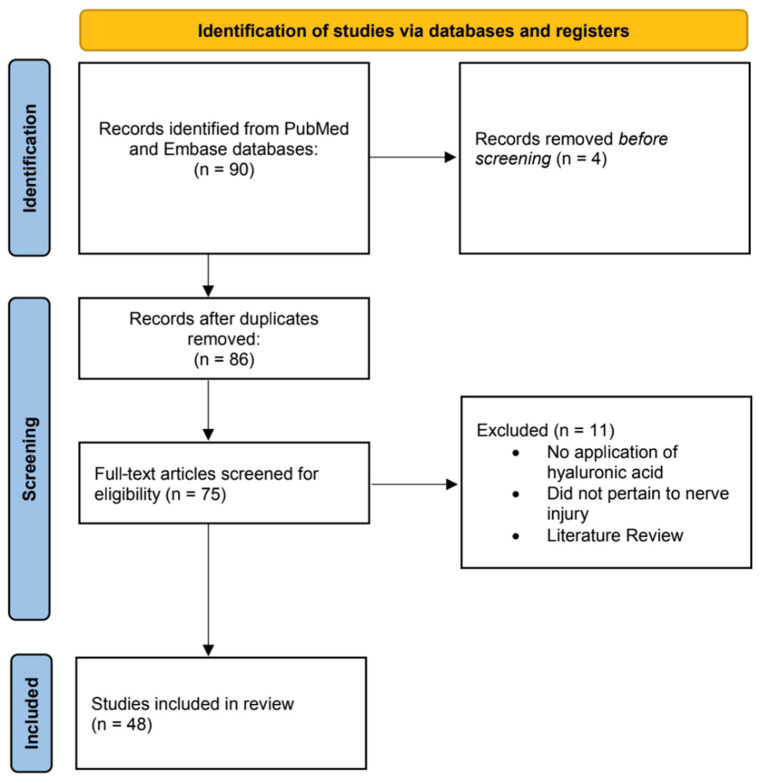
Flow diagram for the systematic review process illustrates the records identified, screened, and included in accordance with PRISMA guidelines.

After removal of duplicates, titles and abstracts were independently screened by two reviewers (C.J.C. and M.S.). Articles meeting preliminary inclusion criteria underwent full-text review. Discrepancies were resolved through discussion and, when needed, consultation with a third reviewer (E.J.H.) and the senior author (B.J.M.). The study selection process is visualized in the PRISMA flow diagram (Figure 1).

### 2.4. Data Extraction

Data extraction was completed independently by two reviewers (C.J.C. and M.S.), with verification by a third reviewer (E.J.H.). Extracted variables included study design, model type (in vitro or in vivo), species, injury type (crush or transection), nerve gap length when specified, hyaluronic acid formulation (native HA, crosslinked HA, composite scaffold, hydrogel, or film), delivery method, adjunctive agents (e.g., exosomes, neurotrophic factors, Schwann cells, pharmacologic agents), follow-up duration, and primary outcomes. Functional outcomes included the Sciatic Functional Index (SFI), compound muscle action potential (CMAP) amplitude, and nerve conduction velocity (NCV). Histologic outcomes included axon count, axon diameter, myelin thickness, and g-ratio. Fibrosis outcomes included collagen deposition, α-SMA expression, and perineural adhesion scoring.

For each in vivo study, the duration of treatment for HA-based formulations was defined as the length of postoperative follow-up during which the HA material remained active in situ or was evaluated for regenerative effect. Because HA hydrogels, films, and composites differ in degradation kinetics, we extracted the full follow-up interval reported in each study as a standardized measure to capture the therapeutic window of each formulation.

### 2.5. Data Synthesis

Because of heterogeneity in study design, biomaterial formulations, animal models, nerve gap lengths, follow-up durations, and outcome reporting, results were synthesized qualitatively. Where three or more studies used similar models and outcomes, findings were summarized narratively to describe trends relative to autografts or conventional repair. A meta-analysis was not performed due to substantial methodological variability.

## 3. Results

### 3.1. Study Overview

This systematic review included 48 studies that explored various biomaterials and intervention techniques for peripheral nerve regeneration (Supplementary Table S1). The studies were conducted across multiple countries, including the United States, China, Germany, and Japan, reflecting a global interest in advancing nerve repair technologies [5,6,7,8,9,10,11,12,18,19,20,22,23,28,29,30,31,32,33,34,35,36,37,38,39,40,41,42,43,44,45,46,47,48,49,50]. The methodologies varied, with most studies utilizing preclinical in vivo models to evaluate outcomes, while a smaller subset focused on in vitro assessments of biomaterial properties and cell compatibility. The studies predominantly utilized rodent models, with Sprague-Dawley and Wistar rats being the most common. A subset of studies used rabbit models to assess scalability and clinical relevance for large nerve defects. Injury models were primarily categorized as crush or transection injuries, which allowed for the evaluation of both spontaneous recovery potential and the need for more complex structural interventions. The biomaterials employed included hyaluronic acid (HA)-based hydrogels, nerve guidance conduits (NGCs), injectable gels, and composite scaffolds [5,8,12,13,14,15,16,18,21,23,32,33,35,36,37,46,51]. Many of these materials were enhanced with bioactive additives such as neurotrophic factors, anti-inflammatory agents, or drug delivery mechanisms to optimize both structural and functional outcomes. Interventions often incorporated innovative technologies such as exosome delivery, photothermal therapy, and magnetic nanoparticle stimulation. Follow-up durations ranged from 2 to 24 weeks, enabling the assessment of both early and long-term outcomes (Supplementary Table S2).

### 3.2. Functional Outcomes

Functional recovery was assessed in all studies using standardized metrics, including the Sciatic Functional Index (SFI), compound muscle action potentials (CMAP), and nerve conduction velocity (NCV) (Supplementary Table S3). SFI scores were consistently used to evaluate walking patterns and nerve recovery. Tang et al. demonstrated that PLGA@Col/HA conduits loaded with exosomes achieved an SFI of −45.6 ± 3.5, nearing autograft levels [24]. These functional improvements were observed at a 12-week follow-up, consistent with the typical regenerative timeframe for rat sciatic nerve models. Similarly, Jafarisavari et al. reported an SFI improvement of −55.3 ± 1.8 in PCL/CH conduits coated with HA and enriched with vitamin B12 and piracetam [22]. This SFI recovery was documented at 8 weeks, the study’s designated endpoint for functional evaluation. These results underscore the potential of these materials to restore functional capabilities comparable to autografts.

Regarding electrophysiological metrics, CMAP amplitudes were frequently used to measure nerve conductivity. Xia et al. reported a CMAP amplitude of 15.8 ± 1.08 mV in their polyacrylamide/HA hydrogel group, which incorporated magnetic nanoparticle stimulation [25]. These electrophysiological gains were measured at 12 weeks, aligning with the hydrogel’s degradation period and its window of bioactivity. Tang et al. observed a significant improvement in latency and peak amplitude in exosome-treated groups compared to untreated controls, highlighting the efficacy of cell-free therapeutic strategies [24]. All electrophysiologic parameters in this study were quantified at 12 weeks, consistent with the study’s predefined late-stage functional endpoint. NCV was also evaluated in several studies, with Liu et al. demonstrating enhanced NCV in soft hydrogel-treated groups compared to stiff hydrogel controls, emphasizing the importance of mechanical properties in biomaterial design [26]. NCV values for the soft-hydrogel group were obtained at 8 weeks, corresponding to the study’s terminal evaluation point.

### 3.3. Structural and Histological Findings

Histological outcomes provided insights into axonal regeneration, myelination, and scar reduction. Axonal regeneration was a key parameter, with studies like Javanmardi et al. reporting superior axonal growth in hydrogel-treated groups containing dexamethasone-loaded HA microparticles [23]. Yang et al. observed significant improvements in axon diameter and density with granular hydrogel conduits, achieving results comparable to autografts [16].

Myelination was another critical focus. Xia et al. demonstrated thicker myelin sheaths and enhanced lamellar organization in Schwann cells treated with magnetically stimulated hydrogels [25]. Similarly, Zhan et al. showed that PDA NPs@HAMA hydrogels significantly improved myelin thickness and axonal alignment while reducing fibrotic tissue [13].

Scar reduction was reported in several studies. Zhan et al. observed a decrease in α-SMA expression and collagen deposition with PDA NPs@HAMA hydrogels, reflecting their anti-inflammatory and anti-adhesion properties [13]. Altinkaya et al. reported reduced fibrosis and enhanced nerve regeneration with subepineural HA injections, further supporting the role of HA-based interventions in minimizing scar formation and promoting nerve repair (Supplementary Table S3) [52].

### 3.4. Comparison of Crush and Transection Models

Crush and transection injury models allowed for a comparative evaluation of biomaterial efficacy (Supplementary Table S2). Crush injuries, characterized by preserved axonal pathways, demonstrated faster recovery with less reliance on complex biomaterials. Kasper et al. highlighted rapid extracellular matrix (ECM) remodeling in crush models treated with HA-based materials, with functional improvements observed as early as two weeks [27].

Transection injuries, in contrast, required robust structural support to facilitate axonal bridging. Jafarisavari et al. reported that PCL/CH conduits coated with HA achieved functional recovery comparable to autografts in a transection model, with enhanced vascularization and reduced fibrosis [22]. Roca et al. utilized multimodular conduits seeded with Schwann cells in a rabbit model, achieving superior outcomes in significant nerve gaps [14]. These studies underscore the importance of tailored biomaterial designs based on the injury type and regenerative requirements.

### 3.5. Temporal Outcomes

Follow-up durations across the studies ranged from 2 to 24 weeks, enabling the evaluation of early and sustained regenerative outcomes (Supplementary Table S4). Short-term studies, such as those by Xia et al., demonstrated rapid improvements in SFI and CMAP metrics within 12 weeks, emphasizing the role of early Schwann cell activation in promoting axonal growth [25]. Long-term studies, such as Roca et al., highlighted the durability of interventions like Schwann cell-seeded conduits, which maintained superior vascularization and myelination outcomes over 24 weeks [14]. These findings suggest that specific biomaterials support early recovery and provide prolonged structural and functional benefits (Supplementary Table S3).

### 3.6. Key Findings and Implications

The findings across the 48 studies demonstrate the versatility and efficacy of HA-based biomaterials in peripheral nerve repair. Functional outcomes, as measured by SFI, CMAP, and NCV, frequently matched or exceeded those achieved with autografts. Histological evaluations revealed significant improvements in axonal regeneration, myelination, and scar reduction. Novel approaches, such as exosome delivery and magnetic nanoparticle stimulation, showed promise for scalable and cell-free therapeutic strategies. These results highlight the clinical potential of these materials, particularly in addressing complex challenges such as large nerve gaps and adhesion prevention. Further research is warranted to optimize these interventions for clinical translation (Supplementary Table S4).

Compared with widely studied conduit materials such as collagen, PCL, and silk fibroin, HA-based scaffolds demonstrated superior anti-adhesion performance and more consistent modulation of the inflammatory microenvironment. While collagen and silk conduits offer excellent structural integrity, their slower degradation and limited control over fibrosis can restrict nerve gliding. PCL conduits provide mechanical support for long gaps but often require biofunctionalization to match the regenerative outcomes observed with HA composites. Several studies in this review showed HA composite conduits achieving autograft-level myelination earlier than PCL or collagen alone, particularly when integrated with exosomes or neurotrophic factors.

### 3.7. Biomaterials and Medical Applications of HA in PNI

Across preclinical models, HA films/wraps reduced adhesions after neurolysis and along repaired nerves, supporting a role as an anti-adhesion barrier [13,18,19,20,21]. HA-containing NGCs and composite hydrogels provided gap bridging and axon guidance, with several platforms approaching autograft-like SFI/CMAP/NCV in short to mid-term follow-up [6,8,12,14,15,16,22,24,25,26]. Injectable HA hydrogels (native or HAMA) served as intraluminal fillers or perineural depots for drug/exosome delivery, improving myelination and reducing α-SMA/collagen in fibrosis assays [6,23,24,25,26,52]. Early clinical series (e.g., adhesion prevention after revision carpal tunnel or neurolysis) report feasibility and safety. Practical applications likely include: (i) adjunct anti-adhesion wraps after neurolysis/complex repairs [6,18,19,20]; (ii) lumen fillers/coatings to biofunctionalize regulatory-cleared conduits [6,8,14,15,16,21]; and (iii) local delivery depots for anti-inflammatory or neurotrophic agents [23,24,25,26,52]. Standardization of HA chemistry, concentration, crosslinking, and dosing is needed before broader clinical adoption [6,11,13].

## 4. Discussion

With its unique biochemical and biophysical properties, hyaluronic acid (HA), a naturally occurring glycosaminoglycan, has become a valuable biomaterial for peripheral nerve repair. Moreover, HA maintains even more functional complexity than traditional biomaterials, more than simply providing structural support; instead, it serves as a regulator of cellular and molecular processes relevant to the regeneration of nerves. This review addresses the mechanistic pathways of HA, explores the discrepancies in these mechanisms between various studies, and discusses the positive estimation and problems associated with HA.

Across the 48 included studies, HA-based biomaterials consistently demonstrated robust improvements in functional, histologic, and electrophysiologic outcomes compared with standard repair techniques, underscoring the translational promise of HA platforms in PNI. Functionally, multiple in vivo models reported SFI scores approaching autograft performance, particularly when HA matrices were supplemented with exosomes, growth factors, or bioactive nanoparticles. For example, Tang et al. and Jafarisavari et al. demonstrated SFI values in the −45 to −55 range, nearly indistinguishable from autograft controls, indicating meaningful restoration of gait mechanics and sciatic nerve function [22,24]. These findings are clinically significant as gait and motor score improvements correlate strongly with meaningful patient recovery milestones such as ambulation and return of functional limb use.

Electrophysiologic outcomes further validated HA’s regenerative effects. Studies showed notable increases in CMAP amplitude and NCV, reflecting enhanced motor-unit activation and signal conduction integrity. Xia et al. reported CMAP amplitudes exceeding 15 mV with magnetically stimulated hydrogels, while Liu et al. demonstrated superior NCV in low-stiffness HA matrices, highlighting mechanobiology as a key design parameter [25,26]. These electrophysiologic results confirm not only axonal regrowth but also functional integration of regenerated fibers, which is essential for clinically durable recovery.

Histologically, HA constructs improved axon density, axon diameter, myelin thickness, and g-ratio across diverse injury models. Notably, granular HA hydrogels and HA-β-TCP composite conduits achieved autograft-level myelination, while dexamethasone-loaded HA microparticles significantly reduced collagen deposition and inflammatory markers [53,54,55,56]. The reduction in α-SMA–positive fibroblasts and organized collagen bands, observed repeatedly across studies, reinforces HA’s anti-adhesion and anti-fibrotic mechanism of action, attributes that are particularly valuable in revision neurolysis and scar-prone surgical fields.

Importantly, the magnitude and consistency of these improvements suggest that HA is not simply a passive scaffold but a bioactive modulator of the nerve microenvironment. The synergy between HA structure, biochemical signaling, and adjunctive payload delivery (e.g., exosomes, NGF) represents a mechanistic strength not shared by many commercial conduits currently used in practice. Innovative delivery strategies such as magnetically templated hydrogels and photothermal-activated HA constructs illustrate HA’s versatility as a tunable therapeutic platform.

HA addresses nerve repair through multiple mechanisms, one of which includes tackling perineural adhesion and scar formation. HA’s potential barriers to functional recovery include the fixed collagen sheaths and scar tissue, which obstruct nerve gliding and axonal regrowth. Several authors have reported ways HA reduces fibroblasts and collagen deposition [13,18]. In their neurolysis models, Shintani et al. showed that sodium hyaluronic acid at a concentration of 1% made a noticeable difference and significantly decreased adhesion-related issues [18]. The functional recovery of the treated nerves was significantly better than that of controls; lower scar thickness and better electrophysiological data reconfirmed these findings. According to Zhan et al., HA methacryloyl hydrogels (HAMA) were combined with photothermal therapy [13]. In contrast to HA treatment alone, this technique more effectively decreased inflammatory responses, reduced fibrous adhesion, and improved HSP72 expression and related inflammation. These findings suggest HA’s anti-fibrotic effects are amplified using bioactive additives or physical stimulation to enhance other effects.

Another mechanism is hyaluronic acid’s (HA) ability to stimulate Schwann cell (SC) activity and axonal growth. SCs are essential in nerve healing, facilitating the regrowth of the axon and myelinating it. Hyaluronic acid interacts with SCs through receptors, including CD44, to facilitate their movement and migration. Huang et al. encapsulated human umbilical cord mesenchymal stem cell exosomes in hyaluronic acid methacrylate (HAMA) hydrogels [6]. Compared to controls, this combination significantly improved SC migration and IL-1β and TNF-α inflammatory markers. Yang et al. employed granular hyaluronic acid (HA) hydrogels in the restoration process [16]. These hydrogels enhanced SC alignment and axon repair more effectively than bulk hydrogels. The best results might be due to the granular structure’s more efficient sharing of nutrients and oxygen.

High-concentration HA also helps provide a hydrated environment, promoting cellular activity and axon growth. Hyaluronic acid scaffolds appear more efficient because they closely resemble the extracellular matrix (ECM), which contains information required for nerve repair [55]. The Huang et al. study damaged cellular membranes, and nerve myelination was induced in rats with collagen fillers, extolling hyaluronic acid–laminin hydrogels (M-HAL) as guide conduits [6]. However, these results were not better than those for autologous grafts, which indicates that HA hydrogels are promising but have a long way to go before reaching their optimum potential. Roca et al. noted the advantages of multinodular designs, which integrate hydroxyapatite (HA) with polylactic acid (PLA) to create polyfunctional conduits [14]. This strategy enhanced Schwann cells’ coverage and vascularization in nerve tissue, making the combination more amenable to treating critical-sized nerve defects than unimodular designs.

HA can creatively deliver neurotrophic agents, anti-inflammatory agents, or stem cells. These combinations benefit its regenerative function and help resolve some challenges associated with nerve repair. Tang et al. highlighted the utilization of exosome-loaded HA-collagen conduits isolated from human umbilical cord mesenchymal stem cells [24]. This Serratia marcescens HA-Ag combination produced moderate levels of vascularization, thicker myelin sheaths, and greater axon diameters comparable to those from autografts. Javanmardi et al. employed a gelatin hydrogel containing dexamethasone-loaded HA microparticles [23]. This approach reduced inflammation levels and improved axonal regrowth, but potentials are not present in hydrogels devoid of bioactive additives. Although HA offers many biochemical benefits, its mechanical properties can be limiting, especially in significant nerve defects where load-bearing conduits are recommended. Due to its low elasticity and fast degradation, rigid structural integrity is automatically compromised. Yan et al. incorporated beta-tricalcium phosphate (β-TCP) and chitosan, which increased the strength of HA-based conduits and were complementary to biocompatibility [15]. Modifying the conduits yielded regeneration results that were as good as those of autografts [15].

Lacko et al. utilized HA hydrogels that had been magnetically templated and possessed a well-aligned 3D microarchitecture [9]. This design improved axonal directionality and Schwann cell invasion, indicating how engineering techniques can rectify HA’s inherent mechanical shortfalls.

The studies underscore the comprehensive role of HA in peripheral nerve repair. However, divergent factors such as the types of animal models, types of injuries, and length of follow-up make it challenging to compare studies directly. For instance, crushed nerves respond better to spontaneous recovery, which may be an overestimate of the efficacy of HA treatments in contrast with transection injuries. Long-gap nerve defects are more challenging to repair and better represent the clinical setting. Here, modular, bioactive, and modulating HA conduits performed better.

In addition to these biologic and structural mechanisms, an important dimension highlighted across several studies is HA’s ability to modulate the biochemical signaling landscape that governs early nerve regeneration. Beyond reducing fibrosis and supporting Schwann cell migration, HA interacts with a range of cell-surface receptors—particularly CD44 and RHAMM, that influence macrophage polarization, angiogenesis, and ECM remodeling [56]. High–molecular-weight HA has been shown to attenuate pro-inflammatory cytokine release and shift macrophages toward an M2-dominant phenotype, creating a more permissive milieu for axonal elongation [57]. This immunomodulatory effect distinguishes HA from many traditional conduit materials that may support cellular infiltration but lack active biologic signaling capacity.

Another area of emerging relevance is the temporal dynamics of HA degradation. Injured peripheral nerve tissue expresses elevated hyaluronidase activity, which alters HA molecular weight distribution and may influence scaffold integrity and signaling properties over time [27]. Kasper et al. demonstrated that hyaluronidase expression peaks at early post-injury timepoints, suggesting that HA-based constructs may require tailored crosslinking or composite reinforcement to maintain functionality during this window of heightened enzymatic activity [27]. This finding underscores the importance of aligning scaffold degradation kinetics with the sequential phases of Wallerian degeneration, Schwann cell proliferation, and axonal regrowth.

A complementary mechanism supported by multiple studies is HA’s role in guiding neovascularization within regenerating nerve tissue. Adequate vascular supply is essential for delivering oxygen, nutrients, and metabolic support to elongating axons. Several HA-containing composites, particularly those incorporating laminin, β-TCP, or aligned microchannels, demonstrated enhanced microvessel density within regenerating nerve bridges [6,14,15]. Improved vascularization was closely associated with thicker myelin sheaths, higher axon counts, and superior electrophysiologic recovery, suggesting that HA indirectly supports regeneration through angiogenic facilitation [14].

There is also growing evidence that HA can influence mechanotransduction processes relevant to nerve healing. Liu et al. showed that low-stiffness HA hydrogels enhanced exosome mobility and Schwann cell infiltration, while higher-stiffness constructs preserved conduit geometry but were less permissive to early cell migration [26]. This highlights a critical trade-off between mechanical robustness and biologic responsiveness. Incorporating HA into composite materials such as cryogel-based conduits, PDLLA/β-TCP/HA blends, or chitosan-reinforced scaffolds has been shown to optimize this balance by preserving mechanical stability while maintaining biologic signaling capacity [58].

Importantly, the literature also identifies several limitations or unintended consequences associated with HA-based systems. For example, Agenor et al. reported that a HA–carboxymethylcellulose formulation applied directly to transected nerves reduced axonal outgrowth in some contexts, suggesting that excessively viscous or occlusive HA layers may impede regeneration if not appropriately engineered [36]. This emphasizes the need for careful control of HA concentration, crosslinking density, and placement relative to fascicular architecture. HA’s utility in clinical translation appears especially promising in the realm of adhesion prevention. Multiple studies in both rodent and rabbit models demonstrated that HA wraps or gels significantly reduced extraneural collagen deposition, preserved nerve glide, and improved functional outcomes after neurolysis or repair [18,19,20,34,54]. These findings align with early human uses of HA-derived anti-adhesion materials in other surgical fields and may represent the most immediate path toward clinical adoption in peripheral nerve procedures.

The potential for HA to function as a biologic carrier remains one of its most compelling translational advantages. HA-based depots have been used to deliver NGF, dexamethasone, mesenchymal stem cell exosomes, vitamin B12-containing cocktails, and small-molecule neuroprotective or anti-inflammatory agents with sustained local release and minimal systemic exposure [2,22,23,24,39,53]. This multifunctionality supports a shift toward integrated “bioactive conduits,” in which HA provides not only structural support and scar modulation but also spatiotemporally controlled delivery of therapeutic payloads.

Future iterations of HA-based scaffolds should incorporate advanced crosslinking strategies such as thiolated HA (SH-HA) and dynamic covalent systems that permit tunable stiffness, shear-thinning injectability, and on-demand degradation. Thiolated HA has been shown to enhance mechanical stability while enabling reversible disulfide bond exchange, offering opportunities for controlled release of neurotrophic factors or exosomes [58,59]. Injectable HA composites, such as HA-gelatin, HA-PEG, or HA-β-TCP hybrids—allow spatially confined delivery and can be engineered to release cues with temporal precision [53]. These next-generation chemistries represent a promising direction for bridging the gap between preclinical efficacy and clinical translation.

## 5. Conclusions

Hyaluronic acid (HA) has shown significant promise as a biomaterial for peripheral nerve repair, demonstrating the ability to reduce scarring, enhance axonal regeneration, and improve functional recovery in preclinical studies. Advancing HA formulations by including bioactive scaffolds and innovative delivery methods could highlight their therapeutic potential. However, to date, there are limited clinical studies and inconsistent experimental methodologies to support its translation into practice. Future research should focus on refining HA applications and conducting clinical trials to bridge the gap between experimental findings and clinical implementation. By addressing these challenges, HA could become a transformative tool in treating peripheral nerve injuries.

## Data Availability

No new data were created or analyzed in this study. Data sharing is not applicable to this article.

## Supplementary Materials

The following supporting information can be downloaded at: https://www.mdpi.com/article/10.3390/jfb17010014/s1, Supplementary File S1, PRISMA checklist. Supplementary Table S1, Animal models and interventions; Supplementary Table S2, Intervention details; Supplementary Table S3, Outcome categories; Supplementary Table S4, Study duration and results.

## Abbreviations

The following abbreviations are used in this manuscript:

HA	Hyaluronic acid
PNI	Peripheral nerve injury
RCT	Randomized controlled trial
NGC	Nerve guidance conduit
HAMA	Hyaluronic acid methacryloyl hydrogel
PLA	Polylactic acid
β-TCP	Beta-tricalcium phosphate
M-HAL	Hyaluronic acid–laminin hydrogel
ECM	Extracellular matrix
SFI	Sciatic Functional Index
CMAP	Compound muscle action potential
NCV	Nerve conduction velocity
SC	Schwann cell

## References

[1] Hussain G., Wang J., Rasul A., Anwar H., Qasim M., Zafar S., Aziz N., Razzaq A., Hussain R., de Aguilar J.-L.G. (2020). Current status of therapeutic approaches against peripheral nerve injuries: A detailed story from injury to recovery. Int. J. Biol. Sci..

[2] Xu H., Yu Y., Zhang L., Zheng F., Yin Y., Gao Y., Li K., Xu J., Wen J., Chen H. (2022). Sustainable release of nerve growth factor for peripheral nerve regeneration using nerve conduits laden with Bioconjugated hyaluronic acid-chitosan hydrogel. Compos. Part B Eng..

[3] Aravamudhan A., Ramos D.M., Nada A.A., Kumbar S.G. (2014). Natural polymers: Polysaccharides and their derivatives for biomedical applications. Natural and Synthetic Biomedical Polymers.

[4] Subramanian A., Krishnan U.M., Sethuraman S. (2009). Development of biomaterial scaffold for nerve tissue engineering: Biomaterial mediated neural regeneration. J. Biomed. Sci..

[5] Whitehead T.J., A Mays E., Prasad M., Mora A., Chen C., Mazhari A., Peduzzi J., Sundararaghavan H.G. (2020). Mechanical, topographical and chemical cues combined with physical therapy for peripheral nerve injuries. Regen. Med..

[6] Huang Z., Kankowski S., Ertekin E., Almog M., Nevo Z., Rochkind S., Haastert-Talini K. (2021). Modified Hyaluronic Acid-Laminin-Hydrogel as Luminal Filler for Clinically Approved Hollow Nerve Guides in a Rat Critical Defect Size Model. Int. J. Mol. Sci..

[7] Dietzmeyer N., Huang Z., Schüning T., Rochkind S., Almog M., Nevo Z., Lieke T., Kankowski S., Haastert-Talini K. (2020). In Vivo and In Vitro Evaluation of a Novel Hyaluronic Acid-Laminin Hydrogel as Luminal Filler and Carrier System for Genetically Engineered Schwann Cells in Critical Gap Length Tubular Peripheral Nerve Graft in Rats. Cell Transplant..

[8] Wu S., Kuss M., Qi D., Hong J., Wang H.-J., Zhang W., Chen S., Ni S., Duan B. (2019). Development of Cryogel-Based Guidance Conduit for Peripheral Nerve Regeneration. ACS Appl. Bio Mater..

[9] Lacko C.S., Singh I., A Wall M., Garcia A.R., Porvasnik S.L., Rinaldi C., E Schmidt C. (2020). Magnetic particle templating of hydrogels: Engineering naturally derived hydrogel scaffolds with 3D aligned microarchitecture for nerve repair. J. Neural Eng..

[10] Jou I., Wu T., Hsu C., Yang C., Huang J., Tu Y., Lee J., Su F., Kuo Y. (2021). High molecular weight form of hyaluronic acid reduces neuroinflammatory response in injured sciatic nerve via the intracellular domain of CD44. J. Biomed. Mater. Res. Part B Appl. Biomater..

[11] Lan S.-M., Yang C.-C., Lee C.-L., Lee J.-S., Jou I.-M. (2017). The effect of molecular weight and concentration of hyaluronan on the recovery of the rat sciatic nerve sustaining acute traumatic injury. Biomed. Mater..

[12] Xuan H., Wu S., Jin Y., Wei S., Xiong F., Xue Y., Li B., Yang Y., Yuan H. (2023). A Bioinspired Self-Healing Conductive Hydrogel Promoting Peripheral Nerve Regeneration. Adv. Sci..

[13] Zhan Y., Zhou Z., Chen M., Gong X. (2023). Photothermal Treatment of Polydopamine Nanoparticles@Hyaluronic Acid Methacryloyl Hydrogel Against Peripheral Nerve Adhesion in a Rat Model of Sciatic Nerve. Int. J. Nanomed..

[14] Roca F.G., Gil Santos L., Roig M.M., Medina L.M., Martínez-Ramos C., Pradas M.M. (2022). Novel Tissue-Engineered Multimodular Hyaluronic Acid-Polylactic Acid Conduits for the Regeneration of Sciatic Nerve Defect. Biomedicines.

[15] Yan X., Wang J., He Q., Xu H., Tao J., Koral K., Li K., Xu J., Wen J., Huang Z. (2021). PDLLA/β-TCP/HA/CHS/NGF Sustained-release Conduits for Peripheral Nerve Regeneration. J. Wuhan Univ. Technol. Sci. Ed..

[16] Yang J., Hsu C.C., Cao T.T., Ye H., Chen J., Li Y.Q. (2023). A hyaluronic acid granular hydrogel nerve guidance conduit promotes regeneration and functional recovery of injured sciatic nerves in rats. Neural Regen. Res..

[17] Du L., Zeng C., Ren X., Li M., Ma R., Gao Y., Xing X., Wang C., Liu Z., Liu Z. (2025). Hyaluronic Acid-Based Therapy for Alleviating Early Lipid Peroxidation in Peripheral Nerve Compression Injury Repair. World Neurosurg..

[18] Shintani K., Uemura T., Takamatsu K., Yokoi T., Onode E., Okada M., Nakamura H. (2018). Protective effect of biodegradable nerve conduit against peripheral nerve adhesion after neurolysis. J. Neurosurg..

[19] Smit X., van Neck J.W., Afoke A., Hovius S.E.R. (2004). Reduction of neural adhesions by biodegradable autocrosslinked hyaluronic acid gel after injury of peripheral nerves: An experimental study. J. Neurosurg..

[20] Ikeda K., Yamauchi D., Osamura N., Hagiwara N., Tomita K. (2003). Hyaluronic acid prevents peripheral nerve adhesion. Br. J. Plast. Surg..

[21] Li R., Liu H., Huang H., Bi W., Yan R., Tan X., Wen W., Wang C., Song W., Zhang Y. (2018). Chitosan conduit combined with hyaluronic acid prevent sciatic nerve scar in a rat model of peripheral nerve crush injury. Mol. Med. Rep..

[22] Jafarisavari Z., Ai J., Mirzaei S.A., Soleimannejad M., Asadpour S. (2024). Development of new nanofibrous nerve conduits by PCL-Chitosan-Hyaluronic acid containing Piracetam-Vitamin B12 for sciatic nerve: A rat model. Int. J. Pharm..

[23] Javanmardi K., Shahbazi H., Hekmat A.S., Khanmohammadi M., Goodarzi A. (2024). Dexamethasone release from hyaluronic acid microparticle and proanthocyanidin-gelatin hydrogel in sciatic tissue regeneration. J. Mater. Sci. Mater. Med..

[24] Tang H., Li J., Wang H., Ren J., Ding H., Shang J., Wang M., Wei Z., Feng S. (2024). Human umbilical cord mesenchymal stem cell-derived exosomes loaded into a composite conduit promote functional recovery after peripheral nerve injury in rats. Neural Regen. Res..

[25] Xia B., Gao X., Qian J., Li S., Yu B., Hao Y., Wei B., Ma T., Wu H., Yang S. (2024). A Novel Superparamagnetic Multifunctional Nerve Scaffold: A Remote Actuation Strategy to Boost In Situ Extracellular Vesicles Production for Enhanced Peripheral Nerve Repair. Adv. Mater..

[26] Liu Z., Tong H., Li J., Wang L., Fan X., Song H., Yang M., Wang H., Jiang X., Zhou X. (2022). Low-Stiffness Hydrogels Promote Peripheral Nerve Regeneration Through the Rapid Release of Exosomes. Front. Bioeng. Biotechnol..

[27] Kasper M.M., Ellenbogen B., Li Y., Schmidt C.E. (2023). Temporal characterization of hyaluronidases after peripheral nerve injury. PLoS ONE.

[28] Zhao X., Fede C., Petrelli L., Pirri C., Stocco E., Fan C., Porzionato A., Tiengo C., De Caro R., Masiero S. (2024). The Impact of Sciatic Nerve Injury on Extracellular Matrix of Lower Limb Muscle and Thoracolumbar Fascia: An Observational Study. Int. J. Mol. Sci..

[29] Ramesh B., Chandrasekaran J., Cherian K.M., Fakoya A.O.J. (2024). Biodegradable nanofiber coated human umbilical cord as nerve scaffold for sciatic nerve regeneration in albino Wistar rats. Folia Morphol..

[30] Yan H., Zhang F., Wang C., Xia Z., Mo X., Fan C. (2015). The role of an aligned nanofiber conduit in the management of painful neuromas in rat sciatic nerves. Ann. Plast. Surg..

[31] Tsuang F.-Y., Chen M.-H., Lin F.-H., Yang M.-C., Liao C.-J., Chang W.-H., Sun J.-S. (2020). Partial enzyme digestion facilitates regeneration of crushed nerve in rat. Transl. Neurosci..

[32] Bhatnagar D., Bushman J.S., Murthy N.S., Merolli A., Kaplan H.M., Kohn J. (2017). Fibrin glue as a stabilization strategy in peripheral nerve repair when using porous nerve guidance conduits. J. Mater. Sci. Mater. Med..

[33] Firat C., Aytekin A.H., Durak M.A., Geyik Y., Erbatur S., Dogan M., Elmas O., Dagli A.F., Celik H. (2016). Comparison of the effects of PRP and hyaluronic acid in promoting peripheral nerve regeneration: An experimental study with vascular conduit model in rats’. Ann. Ital. Chir..

[34] Mekaj A.Y., Manxhuka-Kerliu S., Morina A.A., Duci S.B., Shahini L., Mekaj Y.H. (2017). Effects of hyaluronic acid and tacrolimus on the prevention of perineural scar formation and on nerve regeneration after sciatic nerve repair in a rabbit model. Eur. J. Trauma Emerg. Surg..

[35] Clements B.A., Bushman J., Murthy N.S., Ezra M., Pastore C.M., Kohn J. (2016). Design of barrier coatings on kink-resistant peripheral nerve conduits. J. Tissue Eng..

[36] Agenor A., Dvoracek L., Leu A., Hunter D.A., Newton P., Yan Y., Johnson P.J., Mackinnon S.E., Moore A.M., Wood M.D. (2017). Hyaluronic acid/carboxymethyl cellulose directly applied to transected nerve decreases axonal outgrowth. J. Biomed. Mater. Res. Part B Appl. Biomater..

[37] Meyer C., Wrobel S., Raimondo S., Rochkind S., Heimann C., Shahar A., Ziv-Polat O., Geuna S., Grothe C., Haastert-Talini K. (2016). Peripheral Nerve Regeneration Through Hydrogel-Enriched Chitosan Conduits Containing Engineered Schwann Cells for Drug Delivery. Cell Transplant..

[38] Barreiros V.C.P., Dias F.J., Iyomasa M.M., Coutinho-Netto J., de Sousa L.G., Fazan V.P.S., Antunes R.d.S., Watanabe I.-S., Issa J.P.M. (2014). Morphological and morphometric analyses of crushed sciatic nerves after application of a purified protein from natural latex and hyaluronic acid hydrogel. Growth Factors.

[39] Ziv-Polat O., Shahar A., Levy I., Skaat H., Neuman S., Fregnan F., Geuna S., Grothe C., Haastert-Talini K., Margel S. (2014). The role of neurotrophic factors conjugated to iron oxide nanoparticles in peripheral nerve regeneration: In vitro studies. BioMed Res. Int..

[40] Zor F., Deveci M., Kilic A., Ozdag M.F., Kurt B., Sengezer M., Sönmez T.T. (2014). Effect of VEGF gene therapy and hyaluronic acid film sheath on peripheral nerve regeneration. Microsurgery.

[41] Kim I.G., Piao S., Lee J.Y., Hong S.H., Hwang T.-K., Kim S.W., Kim C.S., Ra J.C., Noh I., Lee J.Y. (2013). Effect of an adipose-derived stem cell and nerve growth factor-incorporated hydrogel on recovery of erectile function in a rat model of cavernous nerve injury. Tissue Eng. Part A.

[42] Park K.-S., Park M.-J., Cho M.-L., Kwok S.-K., Ju J.H., Ko H.-J., Park S.-H., Kim H.-Y. (2009). Type II collagen oral tolerance; mechanism and role in collagen-induced arthritis and rheumatoid arthritis. Mod. Rheumatol..

[43] Torigoe K., Tanaka H.F., Ohkochi H., Miyasaka M., Yamanokuchi H., Yoshidad K., Yoshida T. (2011). Hyaluronan tetrasaccharide promotes regeneration of peripheral nerve: In vivo analysis by film model method. Brain Res..

[44] Slomiany M.G., Dai L., Bomar P.A., Knackstedt T.J., Kranc D.A., Tolliver L., Maria B.L., Toole B.P. (2009). Abrogating drug resistance in malignant peripheral nerve sheath tumors by disrupting hyaluronan-CD44 interactions with small hyaluronan oligosaccharides. Cancer Res..

[45] Magill C.K., Tuffaha S.H., Yee A., Luciano J.P., Hunter D., Mackinnon S., Borschel G.H. (2009). The short- and long-term effects of Seprafilm^®^ on peripheral nerves: A histological and functional study. J. Reconstr. Microsurg..

[46] Zhang H., Wei Y.T., Tsang K.S., Sun C.R., Li J., Huang H., Cui F.Z., An Y.H. (2008). Implantation of neural stem cells embedded in hyaluronic acid and collagen composite conduit promotes regeneration in a rabbit facial nerve injury model. J. Transl. Med..

[47] Özgenel G.Y. (2003). Effects of hyaluronic acid on peripheral nerve scarring and regeneration in rats. Microsurgery.

[48] Özgenel G.Y., Fílíz G. (2004). Combined application of human amniotic membrane wrapping and hyaluronic acid injection in epineurectomized rat sciatic nerve. J. Reconstr. Microsurg..

[49] Özgenel G.Y., Fílíz G. (2003). Effects of human amniotic fluid on peripheral nerve scarring and regeneration in rats. J. Neurosurg..

[50] Adanali G., Verdi M., Tuncel A., Erdogan B., Kargi E. (2003). Effects of hyaluronic acid-carboxymethylcellulose membrane on extraneural adhesion formation and peripheral nerve regeneration. J. Reconstr. Microsurg..

[51] Roche P., Alekseeva T., Widaa A., Ryan A., Matsiko A., Walsh M., Duffy G.P., O’Brien F.J. (2017). Olfactory Derived Stem Cells Delivered in a Biphasic Conduit Promote Peripheral Nerve Repair In Vivo. Stem Cells Transl. Med..

[52] Altinkaya A., Cebi G., Tanriverdi G., Alkan F., Çetinkale O. (2023). Effects of subepineural hyaluronic acid injection on nerve recovery in a rat sciatic nerve defect model. Ulus. Travma Acil Cerrahi Derg..

[53] Davachi S.M., Vazquez M., Soleimani M., Hajmohammadi Z., Mohajer M., Jameie S.B., Khanmohammadi M., Najafi R., Bagher Z., Hassanzadeh S. (2024). Effectiveness of the injectable hyaluronic acid-based microparticles loaded with cannabidiol on rat sciatic nerve injury model. Int. J. Biol. Macromol..

[54] Alsmadi N.Z., Deister C., Evans P., Ghanem T., Smetana B., Mercer D. (2025). Hyaluronate-Alginate Gel-Coated Porcine Small Intestine Submucosa for Nerve Protection Minimizes Extraneural Collagen Deposition in a Preclinical Model. J. Hand Surg. Glob. Online.

[55] Taisescu O., Dinescu V.C., Rotaru-Zavaleanu A.D., Gresita A., Hadjiargyrou M. (2025). Hydrogels for Peripheral Nerve Repair: Emerging Materials and Therapeutic Applications. Gels.

[56] Misra S., Hascall V.C., Markwald R.R., Ghatak S. (2015). Interactions between Hyaluronan and Its Receptors (CD44, RHAMM) Regulate the Activities of Inflammation and Cancer. Front. Immunol..

[57] Rayahin J.E., Buhrman J.S., Zhang Y., Koh T.J., Gemeinhart R.A. (2015). High and low molecular weight hyaluronic acid differentially influence macrophage activation. ACS Biomater. Sci. Eng..

[58] Griesser J., Hetényi G., Bernkop-Schnürch A. (2018). Thiolated Hyaluronic Acid as Versatile Mucoadhesive Polymer: From the Chemistry Behind to Product Developments—What Are the Capabilities?. Polymers.

[59] Summonte S., Racaniello G.F., Lopedota A., Denora N., Bernkop-Schnürch A. (2021). Thiolated polymeric hydrogels for biomedical application: Cross-linking mechanisms. J. Control. Release.

