# Supplementary material for: Mechanistic Advancements and Translational Progress in Hyaluronic Acid-Based Scaffolds and Conduits for Peripheral Nerve Regeneration
Source: J Funct Biomater. 2025 Dec 25;17(1):14. doi: 10.3390/jfb17010014 (PMC12841830; doi:10.3390/jfb17010014)
Supplement: Supplementary file 1 [file jfb-17-00014-s001.zip › jfb-3968341-supplementary.pdf]

Supplementary Table S1. Animal models and interventions.

Author(s), Year	Country	Animal Model (N, Species)	Type of Study (e.g., in vivo, in vitro)	Intervention Types
Zhao et al., 2024	Italy	18 male Sprague-Dawley rats (n = 12 experimental, n = 6 control)	In Vivo	Left sciatic nerve transection injury repaired with an OxPVA-based conduit.
Jafarisavari et al., 2024	Iran	36 male Wistar rats (divided into six groups: 6 per group)	In Vivo and In Vitro	Sciatic nerve injury with a 10 mm gap bridged using nanofibrous nerve conduits fabricated with polycaprolactone (PCL), chitosan (CH), hyaluronic acid (HA), piracetam (PIR), and vitamin B12 (VITB12).
Javanmardi et al., 2024	Iran	24 Sprague-Dawley rats	In Vivo and In Vitro	Sciatic nerve crush injury treated with injectable hydrogels containing dexamethasone-loaded hyaluronic acid microparticles (Dex-HA-Tyr MPs) and proanthocyanidin-gelatin (Gela-PA) hydrogel.
Tang et al., 2024	China	18 female Sprague-Dawley rats, divided into three groups (n=6 each)	In Vivo	Long-gap (10 mm) sciatic nerve defect bridged using PLGA@Col/HA conduits loaded with human umbilical cord mesenchymal stem cell-derived exosomes (hUCMSC-derived exosomes).
Xia et al., 2024	China	36 Sprague-Dawley rats	In Vivo and In Vitro	A 12 mm sciatic nerve defect bridged using a superparamagnetic nanocomposite scaffold (Mag-gel) within a polycaprolactone (PCL) conduit, combined with rotating magnetic field (RMF) stimulation.
Kasper et al., 2023	United States	14 Lewis rats per time point (8 weeks old, male), divided into crush and transection models.	In Vivo	Crush Model: Sciatic nerve crushed 4 mm distal to the greater sciatic foramen for 30 seconds.<br><br>Transection Model: 10 mm sciatic nerve gap bridged with a hollow small intestine submucosa (SIS) conduit.

Xuan et al., 2023	China	Sprague-Dawley rats, n=3 per group	In Vivo and In Vitro	Injectable hydrogel (HASPy) containing HA, cystamine (Cys), and pyrrole-1-propionic acid (Py-COOH), applied to sciatic nerve crush injuries.
Zhan et al., 2023	China	72 male Sprague-Dawley rats, divided into four groups (control, HA, PDA, PDA NPs@HAMA; n=18 per group).	In Vivo	Sciatic nerve adhesion injury treated with photothermal therapy using polydopamine nanoparticles@Hyaluronic acid methacryloyl hydrogel (PDA NPs@HAMA).
Ramesh et al., 2024	India and United States	12 male albino Wistar rats (6 test and 6 control)	In Vivo	Sciatic nerve transection injury repaired using human umbilical cord graft coated with biodegradable poly-L-lactic acid (PLLA) nanofibers.
Altinkaya et al., 2023	Turkey	40 Sprague-Dawley rats, divided into four groups (10 rats each)	In Vivo	Experimental groups included sciatic nerve transection repaired using:<br><br>Primary repair Group 1: 1 cm nerve defect repaired with end-to-end epineural suturing<br><br>Group 2: 1 cm nerve defect repaired with epineural suturing and subepineural hyaluronic acid injection (Group 3).
Xue et al., 2023	United States	40 female Sprague-Dawley rats divided into four groups: sham, suture, fibrin glue, and DNNA (dual-network nerve adhesive).	In Vivo	Sciatic nerve transection repaired using:<br><br>Clinical suture treatment.<br><br>Commercial fibrin glue.<br><br>Dual-network nerve adhesive (DNNA), combining dopamine-isothiocyanate-modified hyaluronic acid (HA-TU-Cat) and decellularized nerve matrix (DPN).

Yang et al., 2023	China	30 male Sprague-Dawley rats divided into five groups (autologous nerve, silicone tube, bulk hydrogel, granular hydrogel, and chitosan conduit).	In Vivo	Sciatic nerve transection with a 10 mm gap bridged using a granular hydrogel conduit.
Liu et al., 2022	China	40 male Sprague-Dawley rats, divided into four groups (Sham, Control, Stiff hydrogel, Soft hydrogel).	In Vivo	Sciatic nerve crush injury treated with hyaluronic acid methacrylate (HAMA) hydrogels of varying stiffness loaded with human umbilical cord mesenchymal stem cell-derived exosomes.
Roca et al., 2022	Spain	12 male rabbits (Oryctolagus cuniculus)	In Vivo	Unimodular conduit (UMC): Single 15-mm hyaluronic acid (HA) module with polylactic acid (PLA) microfibers.<br>Multimodular conduit (MMC): Two 7.5-mm HA modules with PLA microfibers.<br>MMC + hSC: MMC pre-seeded with human Schwann cells (1.25×10^6 cells).
Yan et al., 2021	China	80 male Sprague-Dawley rats, divided into four groups (n=20 per group).	In Vivo	10 mm sciatic nerve defect bridged using:<br>PDLLA (control group)<br>PDLLA/β-TCP/HA/CHS conduits<br>PDLLA/β-TCP/HA/CHS/NGF conduits<br>Autograft (positive control).

Huang et al., 2021,	Germany and Israel	36 adult Sprague-Dawley rats (6 per group across six experimental groups).	In Vivo	15 mm sciatic nerve gap bridged using either:<br><br>Collagen-based NeuraGen® or chitosan-based Reaxon® nerve guides.<br><br>Guides filled with modified hyaluronic acid-laminin hydrogel (M-HAL) at 0.4% or 0.7%.<br><br>Autologous nerve grafts (ANG) as a control.
Tsuang et al., 2020	Taiwan	15 male Sprague-Dawley rats (5 per group).	In Vivo	Sciatic nerve crush injury treated with Liberase-hyaluronic acid (HA) membrane at two concentrations:<br><br>1X (0.001 unit/mm^2)<br><br>2X (0.002 unit/mm^2)
Whitehead et al., 2020	United States	45 adult female Lewis rats, divided into five groups.	In Vivo	Nerve growth conduits (NGCs) with methacrylated hyaluronic acid (MeHA) nanofibers.<br>MeHA nanofibers + glial-cell derived neurotrophic factor (GDNF)-releasing microspheres.<br>MeHA nanofibers + GDNF microspheres + physical therapy (PT).<br>Autograft (control group).
Jou et al., 2021	Taiwan	54 male Sprague-Dawley rats, divided into six experimental groups.	In Vivo	Sciatic nerve crush injury treated with high molecular weight hyaluronic acid (HMW-HA; 3,000 kDa) in a Gelfoam sponge.<br>Groups included control, γ-secretase inhibitor, crush only, crush + HMW-HA, crush + γ-secretase inhibitor, and crush + HMW-HA + γ-secretase inhibitor.

Dietzmeyer et al., 2020	Germany and Israel	64 adult female Lewis rats, divided into nine groups.	In Vivo and In Vitro	Nerve grafts filled with:<br><br>Hyaluronic acid (HA) or Hyaluronic Acid-Laminin (HAL) hydrogel alone.<br><br>HAL hydrogel loaded with naïve Schwann cells (SCs) or fibroblast growth factor-2 overexpressing Schwann cells (FGF2-SCs).<br><br>Control: Autologous nerve graft (ANG).
Wu et al., 2019	United States and China	10 adult Sprague-Dawley rats	In Vivo and In Vitro	10 mm sciatic nerve gap bridged with cryogel-based nerve guidance conduit (NGC).<br>Compared with autografts as the control group.
Lacko et al., 2021	United States	10-week-old Lewis rats (n = 10; 3 experimental groups: non-templated hydrogel, magnetically templated hydrogel, and nerve isograft controls).	In Vivo and In Vitro	10 mm sciatic nerve defect bridged using:<br><br>Non-templated hydrogels.<br><br>Magnetically templated hydrogels with aligned 3D porous architecture.<br><br>Nerve isograft (positive control).
Li et al., 2018	China	60 Sprague-Dawley rats, divided into four groups (control, chitosan, HA, and chitosan+HA; n=15 per group).	In Vivo	Sciatic nerve crush injury treated with:<br><br>Chitosan conduit.<br><br>Hyaluronic acid (HA) injection.<br><br>Chitosan conduit with HA combination.

Shintani et al., 2018	Japan	40 Lewis rats, divided into four groups:<br><br>No-adhesion (neurolysis without adhesion procedure, n=8),<br><br>Adhesion (neurolysis with adhesion procedure, n=12),<br><br>Nerve wrap (neurolysis with nerve conduit, n=12),<br><br>HA (neurolysis with hyaluronic acid, n=8).	In Vivo	Sciatic nerve neurolysis followed by either:<br><br>No additional treatment (adhesion group),<br><br>Wrapping with a flexible biodegradable nerve conduit made of poly(L-lactide) (PLA) and poly(e-caprolactone) (PCL),<br><br>Application of 1% sodium hyaluronic acid (HA).
Roche et al., 2017	Ireland	36 Sprague-Dawley rats (9 per treatment group, including control)	In Vivo	Treatment of 10-mm sciatic nerve gap using a biphasic nerve guidance conduit (NGC) loaded with olfactory-derived stem cells (ONS), with or without nerve growth factor (NGF).
Lan et al., 2017	Taiwan	18 male Sprague-Dawley rats (n = 12 experimental, n	In Vivo	Sciatic nerve crush injury treated with hyaluronan applied via Gelfoam strips of varying molecular weights (350 kDa and 3000 kDa)

		= 6 control)		and concentrations (0.1%, 0.5%, 1.5%).
Bhatnagar et al., 2017	USA	24 female Lewis rats (n = 6 per group)		A 1-cm sciatic nerve gap was bridged using braided porous nerve guidance conduits coated with fibrin glue (FG) or hyaluronic acid (HA), compared to uncoated conduits and reverse autografts.
Firat et al., 2016	Turkey	20 Wistar Albino male rats (18 experimental, 2 donors),Wistar Albino rats.	In Vivo	Group 1 (Autograft): Sciatic nerve defect repaired with an autograft.<br><br>Group 2 (HA): Defect repaired with an allogenic aorta conduit filled with 0.3 mL hyaluronic acid (HA).<br><br>Group 3 (PRP): Defect repaired with an allogenic aorta conduit filled with 0.3 mL platelet-rich plasma (PRP).
Mekaj et al., 2017	Kosovo	30 male European rabbits (Oryctolagus cuniculus), divided into three groups (n = 10 per group)	In Vivo	Topical application of saline (control), hyaluronic acid (HA, 0.5 mL of 16 mg/2 mL solution), or tacrolimus (FK506, 10 ng/mL) wrapped around the sciatic nerve repair site using an absorbable gelatin sponge.
Clements et al., 2016	USA	Female Lewis rats (n = 7 per group for some groups, fewer for others)	In Vivo	Repair of a 1-cm rat sciatic nerve gap using braided nerve conduits with different barrier coatings: uncoated, hyaluronic acid (HA)-coated, electrospun (ES) mat-coated, or a combination of ES and HA coating. Autografts were used as a control group.
Makaj et al., 2015	Kosovo	48 adult male European rabbits (Oryctolagus cuniculus), weight 2.5–3 kg, divided	In Vivo	Sciatic nerve transection and immediate repair with four epineural sutures. The repair site was wrapped with an absorbable gelatin sponge (AGSS) soaked in saline (Group I, control), hyaluronic acid (Group II, 0.5 mL of 16 mg/2 mL), or tacrolimus (Group III, 15 μL of 10 ng/mL FK506 solution).

		into three groups (n = 16 per group).		
Agenor et al., 2017	USA	56 male Lewis rats, divided into three studies:<br><br>Study A: n = 8 per group (HA/CMC, fibrin, empty conduit); Study B: n = 8 per group (HA/CMC wrap, control);Study C: n = 8 per group (HA/CMC wrap, control)	In Vivo	Study A: Sciatic nerve transection repaired with a 5-mm silicone conduit filled with HA/CMC slurry, fibrin, or left empty.<br>Study B: Sciatic nerve transection without repair, with proximal and distal ends wrapped with HA/CMC or left undisturbed.<br>Study C: Sciatic nerve transection with epineurial repair, wrapped with HA/CMC or left untreated.
Meyer et al., 2016	Germany	42 female Wistar rats (n = 6-8 per group depending on the experimental condition)	In Vivo	Reconstruction of a 15-mm critical sciatic nerve defect using hollow chitosan conduits filled with NVR-Gel and genetically engineered Schwann cells (SCs) overexpressing neurotrophic factors (FGF-218kDa or GDNF).
Barreiros et al., 2014	Brazil	40 male Wistar rats, divided into four groups (n = 10 each).	In Vivo	Controlled sciatic nerve crush injury (15 kgf for 10 minutes) treated with:Control (no treatment).Hyaluronic Acid Hydrogel (HAH). Natural latex protein (F1) combined with HAH.

Ziv-Polat et al., 2014	Israel,Germany, Italy	In vitro study using dorsal root ganglia (DRG) explants from Lewis rat embryos.	In Vitro	DRG cultures were exposed to neurotrophic factors (GDNF, βNGF, and FGF-2) either free or conjugated to iron oxide nanoparticles (IONPs) in an NVR-Gel scaffold enriched with hyaluronic acid and laminin.
Zor et al., 2014	Turkey	32 male Sprague-Dawley rats (n = 8 per group)	In Vivo	Sciatic nerve transection and end-to-end neurorrhaphy, with four groups:<br><br>Group I: No additional treatment (control).<br><br>Group II: Nerve coaptation covered with hyaluronic acid (HA) film sheath.<br><br>Group III: Intramuscular injection of VEGF gene plasmid.<br><br>Group IV: Combined HA film sheath and VEGF gene injection.
Kim et al., 2013	South Korea.	Five rats per group, Sprague-Dawley rats.	In Vivo	Application of human adipose-derived stem cells (hADSCs) combined with nerve growth factor (NGF)-incorporated hyaluronic acid-poly(ethylene oxide) (HA-PEO) hydrogel.
Park et al., 2011	South Korea.	40 rats (20 per group), Sprague-Dawley rats.	In Vivo	Application of hyaluronic acid-carboxymethylcellulose (HA-CMC) solution (experimental group) versus saline (control group) around the sciatic nerve repair site.
Torigoe et al., 2011	Japan	4 mice per experimental group, ddY mice.	In Vivo	Application of hyaluronan tetrasaccharide (HA4) solution at varying doses (10, 100, or 1000 μg/mL) directly to transected common peroneal nerve via a film model method.
Slomiany et al., 2009	USA	Not explicitly stated for xenografts, but experiments involved multiple cohorts of nude	In Vivo/ In Vitro	Treatment with small hyaluronan oligosaccharides (o-HA) alone or combined with doxorubicin.

		mice. Nude mice (FOXn1nu strain).		
Magill et al., 2009	USA	30 animals in Phase I (noninjury) and 30 animals in Phase II (injury), Adult male Lewis rats		Placement of Seprafilm (hyaluronic acid-carboxymethylcellulose membrane) on or around sciatic nerves post-surgery in both noninjury (Phase I) and injury (Phase II) models.
Zhang et al., 2008	China	39 rabbits divided into six groups (n ranges from 2 to 11 per group), New Zealand rabbits.	In Vivo	Neural stem cells (NSCs) embedded in a hyaluronic acid (HA)-collagen composite scaffold, supplemented with neurotrophin-3 (NT-3), were implanted to bridge a 5 mm facial nerve gap.
Smit et al., 2004	Netherlands	33 female Wistar rats divided into three injury groups (dissection, crush injury, transection + repair), Wistar rats	In Vivo	Application of autocrosslinked hyaluronic acid (HA) gel (Hyaloglide) to the nerve and surrounding tissues following different types of sciatic nerve injuries.
Özgenel et al., 2004	Turkey	72 rats divided into three groups, Sprague- Dawley rats	In Vivo	Control group: Epineurectomy only.<br>Epineurectomy with human amniotic membrane (HAM) wrapping.<br>Epineurectomy with HAM wrapping and hyaluronic acid (HA) injection inside the HAM envelope.
Özgenel et al., 2003	Turkey	48 rats divided into two groups (experimental and control), Sprague-Dawley rats.	In Vivo	Experimental group: Absorbable gelatin sponge soaked with 0.3 mL of hyaluronic acid (HA) applied around the repair site.<br><br>Control group: Absorbable gelatin sponge soaked with 0.3 mL of saline applied around the repair site.

Ikeda et al., 2003	Japan	20 rabbits, divided into experimental and control groups, Japanese white rabbits.	In Vivo	Control group: Neurolysis without adjuncts.<br>Experimental groups:<br><br>HA group: Neurolysis with hyaluronic acid coating (3 mL applied throughout the procedure).<br><br>Steroid group: Neurolysis with methylprednisolone acetate infiltration at the end of surgery.
Özgenel and Filiz, 2003.	Turkey.	40 rats, divided into experimental and control groups (20 rats each), Sprague-Dawley rats.	In Vivo	Experimental group: Absorbable gelatin sponge soaked with 0.3 mL of human amniotic fluid (HAF) applied around the sciatic nerve repair site.<br><br>Control group: Absorbable gelatin sponge soaked with 0.3 mL of saline applied around the repair site.
Adanali et al., 2003.	Turkey	18 rabbits (9 experimental, 9 control), New Zealand Rabbits	In Vivo	Control group: Epineural repair of sciatic nerve without additional treatment.<br><br>Experimental group: Sciatic nerve repair site wrapped with HA-CMC membrane extending 1 cm proximally and distally

Supplementary Table S2. Intervention details

Author(s), Year	Biomaterial	Mechanism of Action	Nerve Injury Model
Zhao et al., 2024	Hyaluronan	Facilitates gliding movements and acts as a water reservoir, and its reduction post-injury impairs muscle repair and increases stiffness.	Left sciatic nerve transection with a 5 mm gap.
Jafarisavari et al., 2024	Hyaluronic Acid	Hydrates the extracellular matrix, facilitates cell proliferation and migration, promotes axonal growth, and enhances biocompatibility during nerve repair	Complete transection of the sciatic nerve with a 10 mm segment excised.
Javanmardi et al., 2024	Hyaluronic Acid	Sustained dexamethasone release, reduce inflammation, promote cellular migration, and improve tissue stability for sciatic nerve regeneration	Sciatic nerve crush injury using a clamp to create a standard lesion.
Tang et al., 2024	Hyaluronic Acid in collagen sponges	Mimics the ECM, prevents scarring and adhesion, and supports sustained therapeutic release, promoting nerve regeneration and recovery	Complete sciatic nerve transection with a 10 mm gap.
Xia et al., 2024	Hyaluronic Acid (HA) integrated into a polyacrylamide/hyaluronic acid (PAAm/HA) double-network hydrogel	Enhances scaffold hydration, supports cell adhesion, and boosts EV production for nerve repair and regeneration	12 mm sciatic nerve transection in rats.
Kasper et al., 2023	Hyaluronic Acid	Supports cell adhesion, proliferation, and motility, with controlled turnover by hyaluronidases aiding in tissue regeneration and scaffold design for nerve repair	Crush injury to study transient changes in the extracellular matrix (ECM). Transection injury with SIS bridging for studying repair mechanisms.

Xuan et al., 2023	Hyaluronic Acid	Enhances hydration, supports Schwann cell activity via the IL-17 pathway, and promotes nerve regeneration with biocompatible and biodegradable properties	Sciatic nerve crush injury.
Zhan et al., 2023	Hyaluronic Acid Methacryloyl	Reduces peripheral nerve adhesion, enhances HSP72 expression via photothermal effects, and inhibits fibrous tissue formation for improved nerve repair	Sciatic nerve adhesion induced via controlled thermal injury to surrounding muscle tissues.
Ramesh et al., 2024	Hyaluronic Acid	Supports cell adhesion, promotes MSC differentiation, reduces inflammation, and enhances axonal regeneration for nerve repair	Sciatic nerve transection with a 1 cm gap bridged using hUC-WJ graft in the test group and autograft in the control group.
Altinkaya et al., 2023	Hyaluronic Acid	Reduces fibrosis and inflammation, supports epineural structure, and promotes axonal regeneration for improved nerve healing	Sciatic nerve transection with subsequent repair strategies.
Xue et al., 2023	Hyaluronic Acid	Reduces fibrosis, enhances axonal regrowth and remyelination, and supports functional recovery in nerve transection repair by combining bioactive and adhesive properties	Sciatic nerve transection model.
Yang et al., 2023	Hyaluronic Acid	Simulates the ECM, supports nutrient exchange and Schwann cell activity, and promotes axonal growth and functional recovery in nerve repair	Sciatic nerve transection with a 10 mm gap.

Liu et al., 2022	Hyaluronic Acid Methacrylate	Facilitates controlled exosome release, reduces inflammation, and promotes nerve regeneration through enhanced bioavailability and anti-inflammatory effects	Sciatic nerve crush injury induced using vascular forceps for 40 seconds.
Roca et al., 2022	Hyaluronic Acid	Supports cell adhesion, prevents adhesions, reduces glial scar formation, and facilitates axonal growth and vascularization in nerve regeneration scaffolds	Critical-sized sciatic nerve defect (15 mm) in rabbits.
Yan et al., 2021	Hyaluronic Acid	Enhances conduit mechanical properties, supports cell adhesion, enables sustained NGF release, and promotes axonal growth and nerve regeneration	10 mm sciatic nerve defect with conduit implantation or autograft repair.
Huang et al., 2021,	Modified Hyaluronic Acid-Laminin Hydrogel	Supports Schwann cell migration, enhances axonal myelination, and creates a 3D environment for improved nerve regeneration and functional recovery	Acute sciatic nerve transection with a critical defect size of 15 mm.
Tsuang et al., 2020	Hyaluronic Acid	Facilitates Schwann cell mobilization, reduces fibrosis, and promotes axonal regeneration for enhanced nerve repair	Crush injury induced using jewelry forceps to create a standardized lesion, treated with Liberase-HA membranes.
Whitehead et al., 2020	Methacrylated Hyaluronic Acid	Provides aligned nanofiber topography, supports Schwann cell activity, and delivers growth factors for enhanced nerve regeneration	10 mm sciatic nerve gap created by excision.
Jou et al., 2021	High Molecular Weight Hyaluronic Acid	Suppresses inflammation via CD44 signaling, downregulates IL-1β and TLR4, and promotes nerve conduction and motor recovery	Sciatic nerve crush injury.
Dietzmeyer et al., 2020	Hyaluronic Acid-Laminin Hydrogel	Supports Schwann cell migration, delivers neurotrophic factors, and facilitates axonal alignment for nerve	Critical-sized (15 mm) sciatic nerve defect bridged by tubular chitosan-based nerve

		regeneration	grafts.
Wu et al., 2019	Methacrylated Hyaluronic Acid	Enhances cryogel stability, supports Schwann cell adhesion and axonal guidance, and mimics natural nerve tissue for effective nerve regeneration	Sciatic nerve transection with a 10 mm gap.
Lacko et al., 2021	Glycidyl Methacrylate Hyaluronic Acid	Mimics nerve basal lamina, supports Schwann cell migration, and promotes axonal growth through aligned 3D microarchitecture for nerve regeneration	10 mm sciatic nerve gap.
Li et al., 2018	Hyaluronic Acid	Reduces scarring, prevents adhesion, and promotes axonal growth and myelination for effective nerve regeneration	Sciatic nerve crush injury using a needle holder for 30 seconds.
Shintani et al., 2018	Hyaluronic Acid	Prevents scarring and adhesion, enhances nerve gliding, and modulates inflammation to support nerve regeneration after neurolysis	Sciatic nerve adhesion induced by repeated thermal injury to the surrounding muscle after neurolysis.
Roche et al., 2017	Hyaluronic Acid	Supports cell adhesion, enhances axonal growth with laminin and NGF, and provides an optimal environment for nerve repair in a biphasic conduit	10-mm sciatic nerve defect created by transection and bridged with the NGC.
Lan et al., 2017	Hyaluronan	Reduces scarring, activates Schwann cells, and improves nerve conduction, with high molecular weight HA showing superior anti-inflammatory and regenerative effects	Sciatic nerve crush injury sustained for 5 minutes using hemostats.

Bhatnagar et al., 2017	Hyaluronic Acid	Prevent scar tissue infiltration, enhance axonal density and myelination, and support superior functional and muscle recovery in peripheral nerve regeneration	Sciatic nerve transection with a 1-cm gap bridged by nerve guidance conduits.
Firat et al., 2016	Hyaluronic Acid	Reduces scarring, supports axonal regeneration and myelination, and enhances nerve continuity when injected through a vascular conduit	Sciatic nerve defect of 10 mm length created proximally to the bifurcation of tibial and peroneal branches.
Mekaj et al., 2017	Hyaluronic Acid	Prevents scar formation, reduces nerve adherence, and promotes axonal regeneration and myelination, enhancing nerve repair outcomes	Sciatic nerve transection and immediate repair with four epineural sutures under loupe magnification.
Clements et al., 2016	Hyaluronic Acid	Reduce fibrotic infiltration, enhance axonal density and nerve regeneration, and improve functional recovery	Sciatic nerve transection creating a 1-cm gap, bridged using different conduit types or autograft controls.
Makaj et al., 2015	Hyaluronic Acid	Prevents scarring, improves nerve conduction and axonal regeneration, and supports functional recovery when topically applied to nerve repair sites	Right sciatic nerve transection, followed by neurorrhaphy.
Agenor et al., 2017	Hyaluronic Acid-Carboxymethyl Cellulose	Inhibits axonal outgrowth and neuroma formation by creating a temporary blockade at nerve injury sites	Sciatic nerve transection with either repair, unrepaired ends, or epineurial repair.

Meyer et al., 2016	High Molecular Weight Hyaluronic Acid-Laminin Hydrogel	Facilitates Schwann cell delivery and neurotrophic factor expression, promoting axonal regeneration in long-gap nerve defects	Sciatic nerve transection with a 15-mm gap bridged using conduits or autografts (control).
Barreiros et al., 2014	Hyaluronic Acid Hydrogel	Supports axonal regeneration, reduces scar formation, and enhances functional recovery in peripheral nerve injuries, especially when combined with natural latex protein (F1)	Sciatic nerve crush injury (axonotmesis).
Ziv-Polat et al., 2014	Hyaluronic Acid (HA)-Laminin Gel (NVR-Gel)	Stabilizes neurotrophic factors, enhances axonal sprouting, and accelerates myelin formation, supporting peripheral nerve regeneration	Organotypic culture system to mimic peripheral nerve repair processes.
Zor et al., 2014	Hyaluronic Acid	Prevents scar formation, supports axonal elongation, and synergizes with VEGF gene therapy to enhance peripheral nerve regeneration and functional recovery	Sciatic nerve transection followed by epineurial suturing.
Kim et al., 2013	Hyaluronic Acid-Polyethylene Oxide	Supports sustained NGF release, enhances Schwann cell activity, and promotes axonal regeneration and functional recovery in nerve injury models	Bilateral cavernous nerve crush injury (BCNI).
Park et al., 2011	Hyaluronic Acid-Carboxymethylcellulos	Prevents perineural scarring and adhesion, enhances axonal alignment, and supports nerve regeneration when	Sciatic nerve transection and epineurial repair using 10-0 nylon.

	e	applied to repair sites	
Torigoe et al., 2011	Hyaluronan Tetrasaccharide	Enhances axonal outgrowth, accelerates neuronal metabolism, and promotes early nerve regeneration without Schwann cell mediation	Transection of the common peroneal nerve near its bifurcation from the sciatic nerve.
Slomiany et al., 2009	Small Hyaluronan Oligosaccharides	Disrupt CD44 interactions, reduce drug transporter activity, and enhance chemotherapy sensitivity, leading to improved tumor regression in MPNST models	Model system used was not a nerve injury model but xenografts of malignant peripheral nerve sheath tumors (MPNST) in nude mice.
Magill et al., 2009	Hyaluronic Acid-Carboxymethylcellulos e	Reduces perineural scarring and adhesions, improves nerve organization, and supports regeneration by acting as a localized antiadhesive barrier	Phase I: Noninjury model with Seprafilm placed around intact sciatic nerves.<br>Phase II: Sciatic nerve transection followed by repair, with or without Seprafilm wrapping.
Zhang et al., 2008	Hyaluronic Acid-Collagen Composite Scaffold	Supports NSC differentiation, promotes nerve regeneration, and integrates neurotrophic factors for enhanced repair of peripheral nerve defects	Bilateral transection of the facial nerve with a 5 mm defect in rabbits.
Smit et al., 2004	Autocrosslinked Hyaluronic Acid Gel	Prevents scarring, reduces adhesion formation, and supports nerve mobility and functional recovery in peripheral nerve injuries	Dissection, crush injury, and transection with epineural repair of the sciatic nerve.

Özgenel et al., 2004	Hyaluronic Acid	Reduces scar formation, prevents adhesions, and supports nerve gliding for improved peripheral nerve repair.	Circumferential epineurectomy on the right sciatic nerve to simulate scar formation around the nerve.
Özgenel, 2003	Hyaluronic Acid	Prevents scar formation, supports axonal migration, enhances nerve conduction, and reduces adhesion for improved peripheral nerve repair and functional recovery	Sciatic nerve transection and immediate epineural repair with 8/0 monofilament sutures.
Ikeda et al., 2003	Hyaluronic Acid	Reduces scar formation, prevents nerve adhesion, and enhances functional recovery in peripheral nerve repair	Sciatic nerve adhesion induced by thermal coagulation of the neural bed followed by fixation of the nerve to the bed.
Özgenel and Filiz, 2003.	Hyaluronic Acid in Human Amniotic Fluid	Prevents scarring, reduces fibrosis, and supports axonal regeneration and functional recovery through its neurotrophic and anti-inflammatory effects	Sciatic nerve transection and immediate repair with epineural sutures.
Adanali et al., 2003.	Hyaluronic Acid-Carboxymethylcellulose	Reduces adhesion, prevents fibrosis, and enhances axonal regeneration and functional recovery in peripheral nerve repair	Sciatic nerve transection and epineural repair with 10-0 prolene sutures.

Supplementary Table S3. Outcome categories.

Author(s), Year	Functional (Electrophysiological - NCV/CMAP)	Histological	Scar Reduction	Myelination
Zhao et al., 2024	Assessed using the Sciatic Functional Index (SFI); mean score was −87 ± 9.12, indicating severe motor dysfunction.	Immunohistochemistry and Picrosirius Red staining revealed reduced HA and increased collagen levels in the injured and contralateral sides, as well as in the thoracolumbar fascia (TLF).	Not specifically measured.	Observed as increased collagen hindering functional recovery post-reinnervation.
Jafarisavari et al., 2024	Sciatic Functional Index (SFI) improved significantly in the PCL/CH/PIR/VITB12 group (-55.3 ± 1.8 at 12 weeks) comparable to autograft.	H&E, toluidine blue, and immunohistochemical staining for NF200 showed superior axonal regeneration and myelination in the PCL/CH/PIR/VITB12 group.	Minimal inflammation and scarring observed macroscopically and histologically.	Increased axonal growth and myelination detected with NF200 staining in PCL/CH/PIR/VITB12 conduits.
Javanmardi et al., 2024	Sciatic Functional Index (SFI) showed significant improvement in the Dex-HA-Tyr MPs and Gela-PA/Dex-HA-Tyr MPs groups compared to controls.	Enhanced axonal regeneration, reduced vacuolation, and improved myelination observed in treated groups, particularly in the Gela-PA/Dex-HA-Tyr MPs group.	Not explicitly mentioned but implied through reduced fibrosis in treated groups.	Improved thickness and organization of the myelin sheath in the Gela-PA/Dex-HA-Tyr MPs group.
Tang et al., 2024	Improved Sciatic Functional Index (SFI), with the PLGA@Col/HA +	Increased axon diameter and thicker myelin sheaths, similar to autograft group. Enhanced	Reduced collagen deposition in the gastrocnemius muscle as	Improved thickness and organization of the myelin sheath in the exosome-treated group.

	Exosome group nearing autograft efficacy. CMAP showed reduced latency and increased peak amplitude.	vascularization observed via CD31 immunofluorescence.	indicated by Masson trichrome staining.	
Xia et al., 2024	Significant improvement in SFI (-52.0 ± 4.8) and CMAP amplitude (15.8 ± 1.08 mV) in Mag+RMF group, comparable to autografts.	Enhanced axon diameter, myelin sheath thickness, and G-ratio comparable to autograft group.	Lower collagen deposition and muscle atrophy in Mag+RMF group.	Superior myelination with thicker sheaths in the Mag+RMF group.
Kasper et al., 2023	Not explicitly reported.	Immunohistochemical analysis of HYAL1, HYAL2, and CD44, along with HA quantification.	Analysis of ECM remodeling markers (e.g., HA concentration, CD44 expression).	Not explicitly reported but related to ECM changes.
Xuan et al., 2023	Improved SFI (-12.5 ± 1.8% recovery within 30 days), CMAP peak amplitude comparable to control group (6.297 ± 1.774 mV).	Enhanced axon diameter, myelin sheath thickness, and G-ratio, similar to the control group.	Not explicitly mentioned.	Dense myelin lamina observed with TEM; G-ratio similar to the control group.
Zhan et al., 2023	PDA NPs@HAMA group showed improved CMAP amplitude (39.03 ± 1.16	Reduced collagen deposition, lower α-SMA expression, and decreased fibrous tissue density	Significant reduction in gross and histological adhesion scores in the	Increased count of myelinated axons (323.00 ± 5.83) observed in PDA NPs@HAMA group.

	mV) and nerve conduction velocity similar to sham controls.	in the treated group compared to control.	PDA NPs@HAMA group.	
Ramesh et al., 2024	Significant functional recovery observed in walking track analysis, pin-prick test, and sweating quantification by the 4th postoperative week.	Enhanced axonal regeneration, reduced scarring, and consistent G-ratio in both test and control groups.	No evidence of fibrosis or neural scarring in the test group.	Comparable myelin thickness and fiber density between autograft and hUC-WJ grafted nerves.
Altinkaya et al., 2023	Sciatic Functional Index (SFI) values assessed at 0, 2, 4, 8, and 12 weeks. No significant differences in SFI were observed among experimental groups by 12 week	Regenerating myelinated nerve fiber count was significantly higher in Groups 1 and 3 compared to Group 2 ($p<0.05$). Reduced fibrosis was observed in Groups 1 and 3 compared to Group 2.	Group 3 showed decreased perineural adhesion and fibrosis compared to Group 2.	Enhanced regeneration in Groups 1 and 3; Group 3 demonstrated comparable outcomes to primary repair.
Xue et al., 2023	Improved motor and sensory function recovery in DNNA-treated group compared to suture and fibrin glue groups at 10 weeks.	Reduced intraneural fibrosis and inflammation.<br>Enhanced axonal reconnection and directed regrowth.	Minimal scar tissue and better nerve trunk continuity in DNNA group.	Increased myelin thickness and axonal density in DNNA-treated nerves compared to suture and fibrin glue groups.
Yang et al., 2023	Significant improvement in compound muscle action potential (CMAP)	Enhanced axonal diameter, myelin sheath thickness, and more myelinated nerve fibers in	Reduced fibrosis and improved structural recovery compared to bulk	Myelin sheath thickness comparable to autologous nerve repair.

	amplitude and Sciatic Functional Index (SFI) scores for granular hydrogel group, comparable to autograft.	granular hydrogel group.	hydrogel and chitosan groups.	
Liu et al., 2022	Significant improvement in the Sciatic Functional Index (SFI) in the soft hydrogel group (-47.88) compared to the stiff hydrogel group (-79.36) at 14 days post-injury.	Improved axonal alignment and reduced inflammatory markers (IL-1β and TNF-α) in the soft hydrogel group.	Lower macrophage infiltration and fibrosis in the soft hydrogel group.	Not specifically measured.
Roca et al., 2022	Not explicitly reported.	MMC + hSC showed 69 ± 9% of area occupied by myelinated nerve fibers at the central section, compared to 47 ± 14% for MMC and 28 ± 7% for UMC.	Improved vascularization and reduced connective tissue inflammation in MMC + hSC group.	Enhanced alignment and density of myelinated nerve fibers in MMC + hSC.
Yan et al., 2021	Not explicitly reported.	Improved myelin sheath thickness, uniformity, and nerve fiber count in PDLLA/β-TCP/HA/CHS/NGF group, comparable to autograft.	Minimal fibrosis in PDLLA/β-TCP/HA/CHS/NGF group.	Enhanced nerve regeneration with similar outcomes to autografts.
Huang et al., 2021,	Recovery rates (percentage of animals with evocable CMAPs): 100% in ANG group, with	Higher nerve fiber density and better axonal alignment in M-HAL-filled groups than in empty conduits, though not reaching	Not explicitly reported.	Improved myelin thickness observed in M-HAL groups, with comparable outcomes across 0.4% and 0.7% M-HAL concentrations.

	improved recovery in M-HAL-filled groups compared to empty guides.<br>CMAP amplitudes for anterior tibial and plantar muscles showed significant improvements in M-HAL groups compared to empty conduits.	ANG levels.<br>Increased axonal diameter and g-ratio in M-HAL-filled groups compared to controls.		
Tsuang et al., 2020	Sciatic Functional Index (SFI) improved significantly in the 1X group compared to control and 2X groups at 3–6 weeks, plateauing by week 12.	Enhanced Schwann cell mobilization and ECM remodeling observed in the 1X group. The 2X concentration caused excessive tissue digestion, leading to inferior outcomes.	Reduced collagen content in the 1X group compared to controls.	Not explicitly reported.
Whitehead et al., 2020	Improved compound muscle action potential (CMAP) amplitude and reduced latency in GDNF + PT groups.<br>CMAP peak response in GDNF + PT group occurred at lower	Enhanced Schwann cell density and axonal alignment in the GDNF + PT group.<br>Comparable regeneration to autografts.	Reduced connective tissue proliferation observed histologically in GDNF-treated groups	Not explicitly measured.

	stimulation voltage compared to others.			
Jou et al., 2021	Significant improvement in CMAP amplitude and reduced latency in HMW-HA-treated groups compared to untreated and γ-secretase inhibitor groups at 6 weeks.	Reduced IL-1β and TLR4 expression, indicating a suppressed inflammatory response.<br>Downregulation of MMP-9 but not MMP-2 in HMW-HA-treated groups.	Implicit through reduced MMP-9 expression and improved nerve microenvironment.	Not explicitly measured.
Dietzmeyer et al., 2020	ANG showed full recovery with 100% motor function restored. HAL-only groups had partial recovery, with no benefit from SC or FGF2-SC inclusion.	Regeneration parameters (axon count, myelin thickness) were best in ANG, with HAL alone showing moderate success.<br>HAL combined with SCs or FGF2-SCs impaired regeneration.	Not explicitly detailed.	HAL-alone groups demonstrated moderate axonal regeneration and myelination, while HAL + SCs/FGF2-SCs showed diminished outcomes.
Wu et al., 2019	Sensory recovery assessed via Von Frey hair sensitivity, comparable between cryogel and autograft groups.	Comparable density and diameter of myelinated axons in both cryogel and autograft groups.<br>Slightly thinner myelin sheaths in cryogel group compared to autografts.	Not explicitly reported.	Clear lamellar myelin structures observed, with similar axon density to the control group.

Lacko et al., 2021	Not explicitly detailed in this study.	Enhanced axonal density and Schwann cell infiltration in magnetically templated hydrogels.<br>Myelin basic protein staining indicated greater myelination compared to non-templated controls.	Not explicitly discussed.	Observed myelin development comparable to nerve isografts.
Li et al., 2018	Significant improvement in nerve conduction velocity and amplitude in chitosan and chitosan+HA groups compared to controls at 12 weeks.	Increased number and organization of myelinated nerve fibers in chitosan+HA group; reduced extraneural scarring.	Chitosan+HA group showed least connective tissue adhesion compared to other groups.	Enhanced thickness and diameter of myelin sheaths in chitosan+HA group compared to controls.
Shintani et al., 2018	Motor nerve conduction velocity and CMAP amplitude significantly improved in the nerve wrap group compared to the adhesion group, comparable to no-adhesion group.	Reduced perineural scar tissue in the nerve wrap group.<br>Lower macrophage infiltration (CD68+ and CCR7+ cells) in the nerve wrap group compared to adhesion and HA groups.	Lower adhesion scores and biomechanical breaking strength in the nerve wrap group compared to adhesion and HA groups.	Better preservation of axonal morphology and density in the nerve wrap group.
Roche et al., 2017	CMAP improved significantly; ONS-treated groups showed 60% recovery compared to	Axonal count increased with treatment (NGC + ONS = 6,751; NGC + ONS + NGF = 9,925; NGC alone = 4,671). Axon diameter	Not explicitly measured; reduced muscle atrophy observed.	Improved axonal myelination in treated groups.

	21% in controls.	improved by 11.7% with ONS and 26% with ONS + NGF.		
Lan et al., 2017	Improved amplitude and latency of somatosensory-evoked potentials (SSEP), especially with 0.1% 3000 kDa hyaluronan, which showed near-normal recovery by week 6.	Increased axon density and improved nerve fiber structure with 0.1% 3000 kDa hyaluronan compared to other groups.	Reduced fibrosis and anti-inflammatory effects noted, particularly with high molecular weight hyaluronan.	Improved axon diameter and myelin thickness in treated groups.
Bhatnagar et al., 2017	CMAP recovery observed at 12 weeks for HA-coated and uncoated conduits, while no recovery was noted for FG-coated conduits even at 16 weeks. FG-coated conduits showed the lowest recovery (16% gastrocnemius, 15% tibialis anterior), while HA-coated conduits had better recovery (50% gastrocnemius, 60% tibialis anterior).	HA-coated conduits had the highest axonal density (7130 ± 306 #/mm²) and near-normal G-ratio (0.66 ± 0.02), while FG-coated conduits showed poor axonal density (993 ± 854 #/mm²), lowest fascicular area (0.03 ± 0.04 mm²), and highest G-ratio (0.73 ± 0.01).	FG-coated conduits exhibited excessive fibrous tissue infiltration and scar tissue formation.	Enhanced myelination observed in HA-coated conduits and autografts compared to FG-coated conduits.
Firat et al., 2016	Latency: Shorter in PRP group compared to HA group ($p < 0.05$).	Myelination: Group 1 > Group 3 (PRP) > Group 2 (HA). PRP showed improved axonal	Not applicable.	Not applicable.

	Amplitude: Higher in the PRP group, with significant differences between PRP and HA groups ($p = 0.004$). Walk Test Analysis (SFI): PRP group demonstrated better functional recovery compared to HA, with autografts showing the closest recovery to control ($p < 0.05$).	organization and myelin thickness compared to HA. Fibrosis: PRP had lower fibrosis rates than HA, although differences were not statistically significant.		
Mekaj et al., 2017	Gastrocnemius Muscle Mass Ratio (GMMR): HA (0.66) and FK506 (0.69) groups had better muscle preservation compared to saline (0.60).	Reduced scar tissue formation in HA and FK506 groups compared to saline (scar tissue index: 0.67 and 0.68 vs. 0.88, respectively). Better histomorphological organization (graded 4 in HA and FK506 groups vs. 3 in saline group).	Both HA and FK506 significantly reduced scar tissue formation compared to saline.	Enhanced organization of axons and S100 protein expression in HA and FK506 groups compared to saline.
Clements et al., 2016	CMAP amplitude was highest in autografts (37% recovery by week 16), followed by HA-coated conduits (14% recovery). HA-coated conduits	Axon density was highest in HA-coated conduits, followed by uncoated conduits. Fibrous Tissue Infiltration: Minimal in HA-coated conduits; significant in others, especially	HA-coated conduits showed reduced fibrous tissue infiltration, supporting better nerve organization.	G Ratio was smallest in autografts, indicating mature myelination. HA-coated conduits had a moderate G-ratio, suggesting ongoing maturation.

	showed the best electrophysiological outcomes among synthetic conduits.<br>Muscle Mass Recovery: Highest in the autograft group, followed by HA-coated conduits, indicating better functional recovery.	ES-coated.		
Makaj et al., 2015	Motor nerve conduction velocity (MNCV) was significantly higher in HA and FK506 groups compared to saline.<br>By week 12, MNCV recovery reached 51.16% (HA group), 50.42% (FK506 group), and 40.04% (saline group) of the control group values.<br>CMAP amplitudes improved significantly in HA and FK506 groups, reaching ~53% of control by week 12 compared to 38% for saline.<br>Toe-spreading reflex	Not applicable.<br>.	Both HA and FK506 reduced scar tissue formation compared to saline, as inferred from functional outcomes and recovery rates.	Not applicable.

	showed Grade 4 recovery (complete functional restoration) in 81.25% of HA-treated rabbits and 75% of FK506-treated rabbits by week 12, compared to 50% in the saline group.			
Agenor et al., 2017	Not applicable.	Study A: Axonal regeneration significantly inhibited in HA/CMC-filled conduits compared to fibrin-filled or empty conduits.<br>Study B: Wrapping transected nerve ends with HA/CMC almost completely prevented axonal outgrowth.<br>Study C: HA/CMC wrap did not inhibit axonal regeneration when used in conjunction with epineurial repair.	HA/CMC reduced fibrous tissue and aberrant axonal outgrowth in Studies A and B.	Myelinated axon quality was unaffected in regenerating axons in HA/CMC-treated groups.
Meyer et al., 2016	NCV and CMAP recovery was superior in autologous nerve graft (ANG) group, followed by FGF-218kDa SC	Axon Density: ANG had the highest axon density, followed by FGF-218kDa SC conduits.<br>Myelination: G-ratio (indicative of myelin thickness) improved	No significant differences in connective tissue formation or inflammation across groups.	Not applicable.

	conduits (~13% recovery). NCV was significantly faster in ANG-treated animals compared to all other groups.	significantly in ANG and FGF-218kDa groups compared to other treatments.		
Barreiros et al., 2014	Not assessed.	Myelin area/nerve area ratio: Increased significantly in HAH + F1 group after 4 weeks (46.0 ± 9.63) compared to HAH alone and control.<br>Capillary density: Improved vascularization noted in HAH + F1 group compared to HAH alone.	HAH + F1 reduced fibrosis and enhanced nerve bundle organization.	Minimum nerve fiber diameter: Greatest recovery observed in HAH + F1 group (5.35 ± 0.099 μm at 8 weeks).<br>G-ratio: Close to optimal (0.6) in HAH + F1 group, indicating improved myelination.
Ziv-Polat et al., 2014	Not applicable.	Enhanced early axonal outgrowth in cultures treated with conjugated neurotrophic factors compared to free factors.<br>Accelerated onset of myelination was noted with conjugated-GDNF (14 days) compared to free GDNF (20.8 days) or other neurotrophic factors.	Not applicable.	Conjugated-GDNF significantly advanced the onset and progression of myelin formation compared to other treatments.

Zor et al., 2014	Mean peak amplitudes:<br><br>Group I: 4.5 ± 0.6 mV<br><br>Group II: 6.4 ± 0.4 mV<br><br>Group III: 6.7 ± 0.5 mV<br><br>Group IV: 8.5 ± 0.4 mV<br><br>Nonoperated: 9.8 ± 0.5 mV<br><br>Group IV exhibited significantly higher recovery than other groups ($p < 0.001$).	Myelinated axon counts (10 mm distal to the repair site):<br><br>Group I: 105 ± 24<br><br>Group II: 165 ± 19<br><br>Group III: 181 ± 22<br><br>Group IV: 271 ± 23<br><br>Nonoperated: 344 ± 17<br><br>Scar tissue formation significantly reduced in HA-treated groups (II and IV).	Markedly lower connective tissue in HA-treated groups, as confirmed by trichrome staining.	Dense and organized myelin observed in Group IV, with improved alignment of Schwann cells.
Kim et al., 2013	Ratio of intracavernous pressure (ICP) to mean arterial pressure (MAP) upon CN stimulation.	eNOS-positive vessel density, Masson's trichrome staining for collagen/smooth muscle ratio, a-SMA staining for smooth muscle content.	Not directly addressed.	Indirectly inferred through histological improvements in neuronal markers (e.g., beta-III tubulin).
Park et al., 2011	Not reported.	Evaluated scar tissue thickness (scar tissue formation index), fibroblast and inflammatory cell counts, and histomorphological	Significant reduction in perineural adhesion and scar tissue formation index at 12 weeks.	Indirectly assessed through histological organization.

		organization of the nerve repair site.		
Torigoe et al., 2011	Not reported.	Axonal outgrowth assessed by the silver impregnation method and Schwann cell migration visualized by immunohistochemistry (S-100 antibody).	Not applicable.	Not directly addressed, but axonal outgrowth and Schwann cell alignment were evaluated.
Slomiany et al., 2009	Not applicable.	Localization of CD44, BCRP, and Pgp assessed via confocal microscopy.	Not applicable.	Not applicable.
Magill et al., 2009	Functional recovery assessed through biweekly walking track analysis, calculating the Sciatic Function Index (SFI).	Evaluated nerve fiber density, total number of fibers, fiber area, vascularity, and scar tissue formation.	Reduced adhesions qualitatively observed in Seprafilm-treated animals.	Electron microscopy assessed myelinated and unmyelinated fibers, showing no adverse effects of Seprafilm.
Zhang et al., 2008	Assessed via electromyography (EMG) for latency, current threshold, and voltage amplitude.	Morphometric analysis of fiber number, myelin sheath thickness, axon area, and nerve fiber circumference; toluidine blue staining and electron microscopy.	Not explicitly measured but implied through reduced degeneration and fibrosis in scaffold-treated groups.	Assessed via light and electron microscopy, demonstrating improved myelinated fiber alignment in scaffold groups.
Smit et al., 2004	Not measured.	Not directly assessed; adhesion strength inferred biomechanically.	Evaluated by measuring the peak pull-out force required to detach nerves	Not assessed.

			from surrounding tissues.	
Özgenel et al., 2004	Not measured	Assessment of epineurial scar thickness and nerve adherence using an adherence scoring system and scar-tissue formation index.	Macroscopic and microscopic evaluations confirmed reduced scar tissue thickness and adhesions.	Not accessed.
Özgenel et al., 2003	Nerve conduction velocity was significantly higher in the HA-treated group at 12 weeks ($p < 0.05$).	Reduced perineural scar thickness and improved longitudinal organization of regenerating nerve fibers in HA-treated nerves.	Macroscopic and histological evaluation demonstrated significantly reduced perineural scarring in the HA group ($p < 0.05$).	Increased axon and fiber diameter in HA-treated nerves compared to controls ($p < 0.05$).
Ikeda et al., 2003	Latency of compound muscle action potential (CMAP) was measured. HA group showed significantly shorter latency compared to the neurolysis group ($p < 0.05$).	Masson's trichrome staining revealed reduced intraneural and extraneural scar tissue in the HA group compared to other groups.	Tensile strength required to strip the nerve from the neural bed was lowest in the HA group ($p < 0.05$ compared to the neurolysis group).	Not assessed.
Özgenel and Filiz, 2003.	Not directly measured, but Sciatic Function Index (SFI) was evaluated biweekly until 12 weeks. SFI in HAF-treated nerves showed faster and better	Improved axon and fiber maturation, larger axon and fiber diameters ($p < 0.05$) in HAF-treated nerves. Reduced scar tissue thickness and better nerve fiber organization in HAF-treated	Quantitative reduction in perineural scar tissue thickness ($p < 0.05$).	Assessed via axon and fiber diameter, which were significantly improved in HAF-treated nerves.

	recovery compared to saline-treated controls at 10 and 12 weeks ($p < 0.05$).	nerves compared to controls.		
Adanali et al., 2003.	CMAP amplitudes were higher in the experimental group, indicating improved functional recovery. No significant differences in latency were observed.	ncreased myelinated axon count in HA-CMC-treated nerves ($p < 0.01$).<br>Greater axon diameters ($p < 0.05$) and thicker myelin sheaths ($p < 0.01$) in the experimental group.<br>Reduced intraneural and extraneural fibrosis in the experimental group.	Minimal adhesions observed in HA-CMC-treated nerves compared to moderate or severe adhesions in the control group.	Improved axon diameter and myelin sheath thickness in the experimental group.

Supplementary Table S4. Study duration and results.

Author(s), Year	Follow-Up Weeks	Key Findings
Zhao et al., 2024	6 weeks.	➢ HA concentration was significantly reduced, and collagen levels were increased in both the injured and contralateral sides compared to healthy controls.<br>➢ ECM changes in the gastrocnemius muscle and TLF indicated systemic alterations post-injury.<br>➢ These changes in ECM components could exacerbate motor function impairments

		and contribute to musculoskeletal dysfunctions such as low back pain.
Jafarisavari et al., 2024	12 weeks.	➢ PCL/CH/PIR/VITB12 conduits demonstrated regeneration efficiency comparable to autografts in motor and sensory recovery.<br>➢ Enhanced vascularization and reduced fibrosis observed in the experimental group.<br>➢ Functional and structural outcomes supported the potential of these conduits as alternatives to autografts.
Javanmardi et al., 2024	12 weeks.	➢ Gela-PA/Dex-HA-Tyr MPs hydrogel provided superior functional and structural recovery compared to other groups, showing a significant increase in SFI and better histological outcomes.<br>➢ Sustained delivery of dexamethasone minimized inflammatory responses and promoted nerve tissue regeneration.<br>➢ The combined biomaterial demonstrated potential as a therapeutic platform for peripheral nerve repair.
Tang et al., 2024	12 weeks.	➢ PLGA@Col/HA conduits loaded with hUCMSC-derived exosomes significantly improved nerve regeneration and motor function, comparable to autografts.<br>➢ Enhanced angiogenesis and reduced muscle atrophy were observed in the exosome group.<br>➢ The biomaterial demonstrated excellent biocompatibility and safety, showing potential as a cell-free therapeutic option for peripheral nerve repair.
Xia et al., 2024	12 weeks.	➢ Mag-gel with RMF significantly enhanced EV production in vivo, boosting axonal

		regeneration and functional recovery.<br>➢ Morphological and electrophysiological outcomes were similar to autografts, validating the potential of this non-invasive and scalable approach for peripheral nerve repair.<br>➢ Angiogenesis and reduced inflammation in the Mag+RMF group further supported its therapeutic effectiveness.
Kasper et al., 2023	1 day, 3 days, 1 week, 3 weeks, and 6 weeks for crush injury; 3 weeks for transection model.	➢ HYAL1 was significantly downregulated in both injury models compared to uninjured tissue, with peak expression at 3 weeks in the crush model.<br>➢ HYAL2 expression peaked earlier (3 days to 1 week) after crush injury, consistent with its role in initial HA breakdown.<br>➢ CD44 expression was elevated in early phases after crush injury and remained significantly higher in the transection model at 3 weeks, correlating with prolonged ECM remodeling.<br>➢ HA concentration was reduced in the transection model at 3 weeks, indicating delayed tissue stabilization compared to crush injury.
Xuan et al., 2023	4 weeks (30 days).	➢ HASPy hydrogel promoted nerve function recovery and myelin regeneration comparable to the control group.<br>➢ Enhanced Schwann cell function and IL-17 signaling pathway activation contributed to superior nerve regeneration.<br>➢ Demonstrated excellent biocompatibility, conductivity, and self-healing properties for peripheral nerve repair.
Zhan et al., 2023	6 weeks.	➢ PDA NPs@HAMA effectively prevented peripheral nerve adhesion by minimizing

		inflammation and fibrous tissue formation.<br>➢ Improved nerve function and preserved structural integrity were observed, making it a promising material for anti-adhesion and regenerative applications in peripheral nerve repair.
Ramesh et al., 2024	4 weeks.	➢ The hUC-WJ graft demonstrated effective axonal regeneration and functional recovery, comparable to autografts.<br>➢ The use of human umbilical cords, typically discarded as medical waste, presents a viable and ethical alternative to autologous grafts for peripheral nerve repair.
Altinkaya et al., 2023	12 weeks.	➢ Subepineural hyaluronic acid injection (Group 3) improved histological outcomes with reduced fibrosis and enhanced nerve regeneration compared to the epineural repair without hyaluronic acid (Group 2).<br>➢ While functional recovery (SFI) showed no statistical differences, histological findings highlighted the benefits of hyaluronic acid in peripheral nerve repair.
Xue et al., 2023	10 weeks.	➢ DNNA significantly outperformed both suture and fibrin glue treatments in reducing fibrosis, promoting axonal regrowth, and improving functional recovery.<br>➢ The adhesive demonstrated superior biocompatibility and mechanical strength, positioning it as a promising alternative to traditional nerve repair methods.
Yang et al., 2023	16 weeks.	➢ Granular hydrogel outperformed bulk hydrogel and chitosan conduits in functional and structural recovery.<br>➢ Outcomes in the granular hydrogel group were comparable to autologous nerve

		repair, highlighting its potential as an alternative to autografts for peripheral nerve repair.
Liu et al., 2022	2 weeks (14 days).	➢ Soft hydrogels promoted rapid exosome release, which reduced inflammation and supported nerve repair more effectively than stiff hydrogels.<br>➢ The study highlights the importance of hydrogel mechanical properties in peripheral nerve repair.
Roca et al., 2022	24 weeks (6 months).	➢ MMC + hSC provided superior nerve regeneration and myelination compared to MMC and UMC.<br>➢ Multimodular design allowed better vascularization and Schwann cell distribution, offering a promising alternative to autografts for large nerve defects.
Yan et al., 2021	1 and 3 months.	➢ PDLLA/β-TCP/HA/CHS/NGF conduits significantly improved peripheral nerve regeneration compared to controls and were comparable to autograft outcomes.<br>➢ Sustained NGF release enhanced Schwann cell activity and axonal regeneration, suggesting a viable alternative to autografts for peripheral nerve repair.
Huang et al., 2021,	120 days.	➢ M-HAL as a luminal filler improved functional and structural outcomes in both collagen and chitosan-based conduits compared to empty guides.<br>➢ Outcomes did not surpass those of autologous nerve grafts but demonstrated the potential of M-HAL to enhance regeneration in critical defect repair.

Tsuang et al., 2020	12 weeks.	➢ The 1X Liberase-HA membrane significantly accelerated recovery compared to controls and the 2X group, which demonstrated adverse effects due to over-digestion of the ECM.<br>➢ Partial enzymatic digestion can facilitate a more compliant microenvironment, supporting nerve regeneration and earlier functional recovery.
Whitehead et al., 2020	8 weeks.	➢ The combination of GDNF microspheres and PT accelerated functional and structural recovery in peripheral nerve injuries.<br>➢ Gross motor skills and sensory function improved more rapidly in the GDNF + PT group than in other experimental groups.<br>➢ While physical therapy contributed positively to recovery, a synergistic effect with GDNF was not clearly observed, suggesting saturation of neurotrophic factor receptors might limit additional benefits.
Jou et al., 2021	6 weeks.	➢ HMW-HA improved functional recovery and reduced neuroinflammatory mediators.<br>➢ The CD44-ICD pathway played a critical role in regulating the neuroprotective effects of HMW-HA, offering insights into therapeutic applications for peripheral nerve injuries.
Dietzmeyer et al., 2020	120 days.	➢ HAL hydrogel alone supported nerve regeneration but was less effective than autografts.<br>➢ Co-transplanted Schwann cells with HAL reduced regeneration efficacy due to downregulation of neurotrophic factor expression, questioning the combination's

		therapeutic value.
Wu et al., 2019	16 weeks.	➢ Cryogel-based NGC demonstrated effective nerve regeneration and sensory recovery, comparable to autografts.<br>➢ Structural stability and biocompatibility of the cryogel highlight its potential as a substitute for autografts in peripheral nerve repair.
Lacko et al., 2021	2 and 4 weeks.	➢ Magnetically templated hydrogels demonstrated superior axonal growth and Schwann cell infiltration compared to non-templated hydrogels.<br>➢ The architecture mimicking native nerve tissue significantly improved nerve regeneration in the 10 mm gap model, providing a promising off-the-shelf alternative for peripheral nerve repair.
Li et al., 2018	4, 8, and 12 weeks.	➢ Combination of chitosan conduit and HA significantly reduced extraneural scarring and promoted nerve regeneration compared to individual treatments or controls.<br>➢ This synergistic approach provides a promising strategy for peripheral nerve repair and functional recovery.
Shintani et al., 2018	6 weeks.	➢ The PLA/PCL conduit effectively reduced scar formation and adhesion-related damage, preserving nerve function and structure after neurolysis.<br>➢ It outperformed hyaluronic acid in preventing adhesion and maintaining electrophysiological outcomes, making it a promising material for post-neurolysis nerve protection.

Roche et al., 2017	8 weeks	➢ The biphasic NGC with ONS cells significantly improved clinical, electrophysiological, and morphological outcomes, especially with NGF supplementation. ONS cells supported functional recovery and regeneration of axons across the nerve gap, with a 212.5% increase in axon count when combined with NGF.
Lan et al., 2017	8 weeks	➢ High molecular weight (3000 kDa) hyaluronan at low concentration (0.1%) demonstrated superior outcomes in nerve regeneration by reducing pro-inflammatory cytokines (IL-13 and TNF-α), increasing axon density, and improving electrophysiological recovery compared to lower molecular weight (350 kDa) or untreated controls. These results highlight the importance of optimizing hyaluronan properties in peripheral nerve repair.
Bhatnagar et al., 2017	16 weeks	➢ Fibrin glue coatings for porous nerve conduits failed to prevent scar tissue infiltration, resulting in poor axonal regeneration, myelination, and functional recovery. In contrast, hyaluronic acid coatings were more effective in promoting nerve regeneration, mimicking outcomes similar to autografts. The study highlights the limitations of FG as a coating for porous conduits and underscores the potential of HA in peripheral nerve repair.
Firat et al., 2016	Evaluated 12 weeks post-surgery.	➢ Autografts (Group 1) showed the best regeneration and functional recovery, followed by PRP (Group 3) and HA (Group 2).<br>➢ PRP enhanced nerve regeneration more effectively than HA in both functional and histological parameters.<br>➢ PRP demonstrated better outcomes in axonal regeneration and functional recovery

		due to its growth factor-rich composition.<br>➢ HA reduced scar formation but was less effective than PRP in promoting nerve regeneration.<br>➢
Mekaj et al., 2017	12 weeks	➢ Both hyaluronic acid and tacrolimus effectively reduced perineural scar formation and enhanced nerve regeneration compared to saline, with no significant difference between the HA and FK506 groups. These findings highlight the potential of both agents in improving peripheral nerve repair outcomes.
Clements et al., 2016	16 weeks	➢ Braided conduits coated with cross-linked hyaluronic acid showed superior outcomes in nerve regeneration compared to uncoated and ES-coated conduits. HA-coated conduits reduced fibrous tissue infiltration, supported organized nerve regeneration, and improved functional outcomes, approaching those of autografts. ES-coated conduits, despite initial promise, facilitated fibrous infiltration, reducing their effectiveness. These results highlight the critical role of barrier coatings in optimizing nerve conduit performance.
Makaj et al., 2015	12 weeks	➢ Hyaluronic acid and tacrolimus were equally effective in enhancing nerve regeneration and functional recovery after sciatic nerve repair. Both agents improved MNCV, CMAP amplitude, and motor function recovery compared to saline, with minimal differences between the HA and FK506 groups. This study underscores the potential of both HA and FK506 as effective treatments to improve outcomes in peripheral nerve repair.

Agenor et al., 2017	4 weeks	➢ Direct application of HA/CMC to transected nerve ends or within a conduit significantly inhibited axonal outgrowth, demonstrating its potential for neuroma management by acting as a barrier to aberrant axonal regeneration. However, when used with epineurial repair, HA/CMC did not impede normal axonal regeneration, indicating its selective inhibitory effect is dependent on the context of application.
Meyer et al., 2016	17 weeks	➢ Chitosan conduits filled with FGF-218kDa-overexpressing SCs demonstrated enhanced axonal regeneration and functional recovery, but performance remained inferior to autologous nerve grafts. The study underscores the potential of combining engineered Schwann cells and hydrogels for critical-length nerve defect repair.
Barreiros et al., 2014	8 weeks	➢ The combination of hyaluronic acid hydrogel and natural latex protein showed superior outcomes in nerve regeneration compared to individual treatments, enhancing myelination, vascularization, and fiber organization. These findings highlight the potential of this biomaterial combination in peripheral nerve repair.
Ziv-Polat et al., 2014	Observations up to 4 weeks in culture.	➢ Conjugation of neurotrophic factors (especially GDNF) to iron oxide nanoparticles significantly improved their stability, bioavailability, and efficacy in promoting early nerve regeneration and myelination in DRG cultures. Conjugated-GDNF demonstrated the greatest impact on accelerating myelin formation, making it a promising candidate for future peripheral nerve repair strategies using bioartificial nerve grafts.

Zor et al., 2014	4 weeks	➢ The combination of VEGF gene therapy and HA film sheath significantly enhanced nerve regeneration, demonstrated by superior electrophysiological recovery, increased axon counts, and reduced scar formation. This synergistic approach addressed both the cellular (VEGF) and noncellular (HA) aspects of nerve repair, offering a promising strategy for peripheral nerve regeneration.
Kim et al., 2013	Four weeks post-intervention.	➢ The hADSC/NGF-hydrogel group showed significant improvement in erectile function (ICP/MAP ratio) compared to other groups.<br>➢ Histological analysis revealed higher endothelial nitric oxide synthase (eNOS) expression and better smooth muscle preservation in the corpus cavernosum.<br>➢ Collagen/smooth muscle distribution normalized in the hADSC/NGF-hydrogel group, suggesting effective prevention of smooth muscle atrophy.<br>➢ The hydrogel facilitated sustained NGF release and enhanced hADSC engraftment into the CN.
Park et al., 2011	3, 6, 9, and 12 weeks post-operation.	➢ The experimental group showed significantly reduced perineural adhesion and fibroblast/inflammatory cell counts compared to the control group ($p < 0.05$).<br>➢ Scar tissue formation index and histomorphological organization scores were significantly better in the experimental group at 12 weeks ($p < 0.05$).<br>➢ HA-CMC solution effectively reduced scarring and promoted better alignment of regenerating axons at the repair site.<br>➢ No adverse effects such as inflammatory reactions or wound healing impairment were observed.

Torigoe et al., 2011	Assessed at intervals from 6 hours to 4 days post-intervention.	➢ HA4 (100 µg/mL) significantly enhanced axonal outgrowth compared to control and other doses ($p < 0.01$).<br>➢ Axonal outgrowth rate with HA4 (100 µg/mL) was 268 µm/day initially, approximately 3 times faster than control (77 µm/day).<br>➢ Schwann cells appeared on day 3 post-axotomy, but their behavior and distribution in HA4-treated nerves were similar to controls.<br>➢ No promotion of axonal outgrowth was observed with intraperitoneal administration of HA4.
Slomiany et al., 2009	Monitored tumor growth and drug responses over 2 weeks.	➢ HA treatment disrupted CD44-transporter complexes, reduced drug efflux, and decreased doxorubicin resistance in MPNST cells in vitro.<br>➢ Systemic administration of o-HA (0.5–5 mg/kg) inhibited tumor growth, with higher doses (5 mg/kg) inducing regression in vivo.<br>➢ Combined treatment with suboptimal doses of o-HA (0.5 mg/kg) and doxorubicin (1 mg/kg) synergistically induced tumor regression, outperforming additive effects of the individual treatments.<br>➢ o-HA exhibited no significant toxicity or immunogenicity.
Magill et al., 2009	18, 32, and 42 days post-intervention in Phase II; 45 and 90 days in Phase I.	➢ Phase I: No differences in histological outcomes or functional deficits between Seprafilm and control groups. Reduced scar formation was qualitatively noted.<br>➢ Phase II: Seprafilm-treated animals had a greater number of nerve fibers at 18 days post-repair ($p < 0.05$), but non-Seprafilm animals exhibited higher fiber density and nerve percentage at 42 days ($p < 0.05$).<br>➢ Functional outcomes (SFI) were equivalent between groups, suggesting no detrimental effects of Seprafilm on nerve regeneration.<br>➢ Seprafilm reduced perineural scar tissue formation without compromising

		revascularization or causing inflammation.
Zhang et al., 2008	12 weeks post-implantation.	➢ NSC-embedded NT-3-supplemented HA-collagen scaffolds demonstrated the most significant nerve regeneration, with EMG parameters approaching normal levels by 12 weeks.<br>➢ Histological findings showed better fascicle organization, reduced degeneration, and near-normal myelin thickness in scaffold-treated animals.<br>➢ Functional recovery (e.g., blink reflex and ear movement) was observed in scaffold-treated rabbits but remained incomplete compared to controls.<br>➢ No signs of immunogenicity or inflammation were noted in scaffold-treated animals.
Smit et al., 2004	6 weeks post intervention	➢ The HA gel significantly reduced adhesion strength in all injury types:<br>➢ Dissection group: 26% reduction in pull-out force ($p = 0.015$).<br>➢ Crush injury group: 29% reduction ($p < 0.01$).<br>➢ Transection + repair group: 38% reduction ($p < 0.01$).<br>➢ No adverse effects, such as infection or wound dehiscence, were observed in any treated animals.<br>➢ The biomechanical model provides a reliable and quantitative method to evaluate adhesion-reduction therapies in peripheral nerve injuries.
Özgenel et al., 2004	Evaluation at 4 and 12 weeks post-surgery.	➢ Combined HAM wrapping and HA injection (Group 3) showed the least adhesion and perineurial scar tissue formation compared to other groups ($p < 0.05$).<br>➢ Group 3 demonstrated significantly improved nerve gliding and lower adhesion scores compared to Group 2 (HAM wrapping only).<br>➢ Histological analysis confirmed that Group 3 had the thinnest scar layer and minimal

		connective tissue compared to Groups 1 and 2.<br>➢ HAM and HA are safe and effective for preventing adhesions in peripheral nerve injuries.
Özgenel et al., 2003	Evaluations at 4 and 12 weeks post-intervention.	➢ HA significantly reduced perineural scar formation and improved nerve regeneration parameters, including conduction velocity, axon diameter, and longitudinal organization of nerve fibers.<br>➢ Functional recovery, measured by Sciatic Function Index (SFI), was faster and better in the HA group, with significant improvement observed at 10 and 12 weeks ($p < 0.05$).<br>➢ Gastrocnemius muscle mass ratio was higher in HA-treated animals, reflecting better preservation of muscle innervation.
Ikeda et al., 2003	Evaluated at 6 weeks after the neurolysis procedure.	➢ HA coating during neurolysis significantly reduced scar formation and adhesion compared to steroid or neurolysis alone.<br>➢ HA group demonstrated improved electrophysiological function with shorter CMAP latency and less intraneural and extraneural scarring.<br>➢ HA outperformed steroids in reducing tensile strength of adhesions, indicating reduced nerve tethering.<br>➢ Early application of HA in the surgical field was critical for preventing adhesion.
Özgenel and Filiz, 2003.	Evaluations conducted at 4 and 12 weeks post-surgery.	➢ HAF significantly reduced perineural scarring and enhanced nerve regeneration.<br>➢ Axon and fiber diameters, along with organization at the repair site, were significantly better in HAF-treated nerves.<br>➢ Functional recovery (SFI) was faster and superior in the HAF group compared to controls.

		➢ HAF showed no signs of inflammatory reactions, indicating safety for topical application.
Adanali et al., 2003.	Improved axon diameter and myelin sheath thickness in the experimental group.	➢ HA-CMC membrane significantly reduced extraneural and intraneural scar formation and improved axonal regeneration.<br>➢ Nerves treated with HA-CMC demonstrated better morphometric parameters (axon count, axon diameter, and myelin thickness).<br>➢ HA-CMC-treated nerves showed minimal adhesions and better functional recovery, as evidenced by higher CMAP amplitudes.<br>➢ HA-CMC is a safe, biocompatible, and effective material for preventing scarring and enhancing nerve regeneration
